# Economic evaluation of alternative hepatitis C treatment options: a post hoc analysis of the VIETNARMS trial

**DOI:** 10.1016/j.eclinm.2026.103969

**Published:** 2026-05-07

**Authors:** Hugo C. Turner, Sayara Ahmed, Huyen Anh Nguyen, Le Manh Hung, Jennifer Van Nuil, Dang Trong Thuan, SEARCH investigators, Azim Ansari, Eleanor Barnes, Dang Thi Bich, Dao Bach Khoa, Jeremy N. Day, Barnaby Flower, Leanne McCabe, Richard M. Hoglund, Evelyne Kestelyn, Cherry Kingsley, Chau Le Ngoc, Le Thanh Phuong, Le Thi Thao, Nguyen Bao Tran, An Nguyen Chau, Nguyen Kim Tuyen, Nguyen Ngoc Phuong, Sarah Pett, Pham Ngoc Thach, Motiur Rahman, David Smith, Joel Tarning, Rogier Van Doorn, Vo Minh Quang, Vo Thi Thu, Vu Kim Hang, Vu Thu Huong, Nguyen Thanh Dung, Guy E. Thwaites, Ann Sarah Walker, Nguyen Van Vinh Chau, Graham S. Cooke, Timothy B. Hallett

**Affiliations:** a MRC Centre for Global Infectious Disease Analysis, School of Public Health, Imperial College London, Norfolk Place, London, United Kingdom; b Oxford University Clinical Research Unit, Wellcome Trust Major Overseas Programme, Ho Chi Minh City, Vietnam; c Hospital for Tropical Diseases, Ho Chi Minh City, Vietnam; d Centre for Tropical Medicine and Global Health, Nuffield Department of Medicine, University of Oxford, Oxford, United Kingdom; e MRC Clinical Trial Unit at UCL, University College London, United Kingdom; f Department of Infectious Disease, Imperial College London, United Kingdom

**Keywords:** Hepatitis C, DAA, Economic evaluation, Cost, Cost-minimization analysis, Treatment strategies

## Abstract

**Background:**

Hepatitis C remains a leading cause of liver disease worldwide, and access to Direct-Acting Antiviral (DAA) treatment remains limited in many settings. Alternative treatment strategies that require fewer tablets and clinical visits could help improve equitable access, and new approaches have recently been found to be non-inferior in producing sustained viral suppression.

**Methods:**

We did a cost-minimization analysis of alternative treatment options for non-cirrhotic patients evaluated in the VIETNARMS trial (ISRCTN61522291), conducted between 19/06/2020 and 10/05/2023 in Vietnam. These were: (i) ‘response guided’ (which adjusts treatment duration based on 1-week viral load); (ii) ‘induction maintenance’ (which reduces the dosing frequency in later weeks of treatment); and (iii) ‘Peg-IFN + DAA’ (4 weeks of DAAs combined with four weekly doses of PEGylated interferon (Peg-IFN)). The primary outcome was the cost per cure. A disaggregated societal perspective was adopted, including stratification for the healthcare provider and patient costs.

**Findings:**

The three alternative treatment strategies were projected to have lower costs per cure than standard 12-week DAA treatment in the base-case scenario: US$202 (15%) less for ‘response guided’, US$234 (18%) less for ‘induction maintenance’, and US$163 (12%) less for ‘Peg-IFN + DAA’. However, the potential for cost savings, and which strategy had the lowest cost per cure, depended on the assumed cost of DAA drugs: the strategy with the lowest cost per cure was generally ‘induction maintenance’ when DAA drug costs for a standard treatment course were under US$1000, but Peg-IFN + DAA when DAA costs exceeded US$1500. In some scenarios, lower costs per cure were achieved through reduced health system expenditures, despite increased costs to patients.

**Interpretation:**

Alternative strategies for Hepatitis C treatment could reduce costs for providers and patients. As this is highly dependent on the variable costs of DAAs, approaches should be fit carefully to the prevailing context.

**Funding:**

Wellcome Trust, Medical Research Council.

**Research in context**

**Evidence before this study**

We searched PubMed, Embase, the Cochrane Library, and EconLit for studies published between Jan 1, 2000, and Dec 31, 2023, using the terms “hepatitis C”, “direct-acting antivirals”, “pegylated interferon”, “treatment strategy”, “shortened duration”, “response-guided”, “cost”, and “economic evaluation”. No language restrictions were applied, and reference lists of relevant papers were screened. Previous evaluations consistently showed that direct-acting antivirals (DAAs) achieve cure rates above 90% with standard durations and are more cost-effective than solely interferon-based regimens (i.e. interferon without DAAs) in high-income settings. A limited number of studies have examined the cost-effectiveness of shortened DAA regimens, which typically have lower cure rates or have been tested only in highly restricted patient populations. However, nearly all such evaluations have been conducted in high-income countries, with limited generalizability to low- and middle-income settings. Crucially, no prior economic evaluations have incorporated efficacy data directly generated from the VIETNARMS trial in Vietnam, which provides the locally relevant clinical outcomes underpinning this study.

**Added value of this study**

To our knowledge, this is the first comprehensive economic evaluation of alternative hepatitis C treatment strategies in Vietnam, a lower-middle-income country with high hepatitis C burden. We assessed three strategies aimed at reducing total DAA exposure compared with the standard recommended 12-week daily treatment, using locally derived costs and effectiveness data. By exploring a broader range of treatment adaptations, we provide novel insights into potential approaches that could expand access to curative therapy in resource-limited settings.

**Implications of all the available evidence**

While standard full-course (12-week) DAA therapy remains highly effective, alternative approaches such as shortened or response-guided regimens could offer cost savings and improve affordability for national hepatitis C programmes, as well as expand access to hard-to-reach populations. These strategies may promote more equitable treatment coverage in health systems with constrained fiscal space. Nevertheless, the extent of potential cost savings depends critically on DAA unit prices and other treatment-related costs, which vary widely across and within countries. The financial burden of each strategy falls differently on healthcare providers and patients. Some strategies (particularly Peg-IFN + DAA) reduced health system costs whilst simultaneously increasing patient out-of-pocket burdens. Policy decisions should therefore account for how costs are shared between health systems and patients and for implementation barriers that could create inequities. This highlights that policy recommendations should adapt to the local context and the importance of not using a one-size-fits-all approach.

## Introduction

Hepatitis C is a viral infection (HCV) that remains one of the leading causes of liver disease worldwide and is responsible for a substantial global disease burden.^1^ In 2016, the World Health Organization (WHO) proposed eliminating hepatitis C as a public health threat by 2030.^2^

The development of direct-acting antiviral (DAA) drugs has revolutionized hepatitis C treatment.^3^ Access to DAAs has grown in recent years, but nevertheless remains limited in many settings: of the 58 million persons living with HCV infection globally in 2019, an estimated 15.2 million (21%) knew their diagnosis, and of those diagnosed with chronic HCV infection, around 9.4 million (62%) persons had been treated with DAAs by the end of 2019.^4^

One barrier to improved treatment availability is the cost of DAAs, which varies notably across different countries.^5^^,^^6^ With the introduction of generic versions of DAAs, costs have dramatically decreased, especially in low-income and lower-middle-income countries.^4^^,^^5^ However, DAA drugs remain expensive in many high- and some upper-middle-income countries.^4^, ^5^, ^6^ Furthermore, even among low-income and lower-middle-income countries, costs can vary significantly (costs ranging between US$39 and US$4050 across countries in Supporting Figure S1,^7^),^5^^,^^8^ and the total funding available for hepatitis programs is a small fraction of that estimated to meet the need.^9^

Another barrier is the significant demands a 12-week regimen places on patients. Depending on local insurance and funding arrangements, patients can have substantial out-of-pocket expenditures and can also incur productivity costs (e.g., missing work and caring responsibilities) when attending care.^10^, ^11^, ^12^

Therefore, there is a pressing need to develop and evaluate alternative strategies for hepatitis C treatment that reduce overall cost and minimize patient burden. The VIETNARMS trial in Vietnam^13^ therefore tested the effectiveness of alternative strategies:

1. Response-guided: Tailoring DAA treatment length to 4, 8 or 12 weeks' DAAs based on a viral load test 7 days after starting treatment.
2. Induction-maintenance: 2 weeks' daily therapy (induction) followed by 10 weeks taking DAAs 5 days per week with weekends off (maintenance).
3. Peg-IFN + DAA: 4 weeks' DAAs combined with four weekly doses of PEGylated interferon (Peg-IFN).

The trial demonstrated that all three approaches were non-inferior to the standard 12-week DAA course using a 10% non-inferiority margin on the sustained virological response at 12 weeks (SVR12)^13^ Therefore, here, we performed a cost-minimization analysis of these alternative treatment strategies in Vietnam.

## Methods

We conducted a cost-minimization analysis of the three novel strategies in comparison to a standard treatment of 12-weeks daily DAAs comparator. Cost-minimization is the approach framework as we assumed that in the case of initial treatment failure, the patient would receive one round of retreatment (12 weeks’ daily DAAs using an alternative regimen plus ribavirin), which would be successful in achieving SVR12. Therefore, we assumed that differences in long-term health outcomes between the treatment strategies would be negligible, as all patients were expected to be cured either by their initial regimen or, if required, by a single round of retreatment. Under this assumption, the overall health impact would be equivalent across strategies, leaving cost as the distinguishing factor (justifying the use of a cost-minimization analysis^14^).

The modeling approach was based on a projection of the cost per cure of the different strategies, accounting for the unit cost of a treatment strategy as well as the costs incurred for that proportion that need to receive a round of retreatment. The calculations were performed in Excel based on the following equation:

Cost per cure of the strategy_i_ = Unit cost of the strategy_i_ ∗ Effectiveness of the strategy_i_ + Unit cost of retreatment ∗ (1-Effectiveness of the strategy_i_).

This modeling approach was used to simulate a hypothetical population of non-cirrhotic patients seeking treatment at the Hospital for Tropical Diseases (HTD) in Ho Chi Minh City, the primary hospital responsible for HCV treatment in Southern Vietnam. The care of the patients (type and number of tests) was assumed to follow Vietnam's 2021 treatment guidelines for hepatitis C.^15^

The time horizon of the analysis accounted for a first-line and, in the case of treatment failure, one potential round of retreatment (a total of 48 weeks). The CHEERS checklist^16^ was followed for reporting in this manuscript.

### Effectiveness of the strategies

The relative effectiveness of the different strategies was based on the outcomes of the VIETNARMS trial,^13^ specifically that SVR12 (i.e cure) relative to standard treatment (12-weeks daily DAAs) was −5.7% (90% Credible intervals (CrIs): −9.6%, −2.3%), +0.6% (90% CrIs: −1.1%, 2.7%) and −4.5% (90% CrIs: −8.3%, −1.3%), for Response-guided, Induction-maintenance, and Peg-IFN + DAA strategies respectively (Supporting Table S1). The VIETNARMS trial,^13^ was conducted in Vietnam between 2019 and 2022, and enrolled 624 non-cirrhotic patients with chronic HCV at the HTD in Ho Chi Minh City and the National Hospital for Tropical Diseases in Hanoi. We considered aggregate effectiveness values (rather than stratifying by HCV genotype or (randomized) use of sofosbuvir/daclatasvir versus sofosbuvir-velpatasvir as the trial found no evidence of heterogeneity/interaction by these or any other factors).

### Cost assumptions

The costs were estimated within the context of HTD in Ho Chi Minh City, the primary hospital responsible for HCV treatment in Southern Vietnam. The analysis was conducted from a disaggregated societal perspective,^17^ with costs stratified into the following categories: “Direct medical costs–medical services and monitoring”, “direct medical costs–drug costs”, and the costs for patients accessing the treatment (“direct non-medical and productivity costs”).^18^ The costs were also stratified depending on whether they were incurred by the healthcare provider (the costs incurred by the national health insurance program) or the patient.^17^ The costs are presented in 2023 US$ costs (US$1 = VND23,787).^19^ Because the time horizon was less than one year, no discounting was applied.

Severe adverse reactions were rare across all strategies investigated^13^ and the majority of the reported adverse reactions were associated with Peg-IFN + DAA. The majority of adverse events were identified either at the weekly symptom review or via routine blood tests and did not require additional clinic visits. We therefore assume that the medical and visit costs associated with adverse events would be predominantly captured. However, there is potential for lost productivity from adverse events that are not captured, and this is considered in an additional scenario analysis.

#### Direct medical costs–drugs

The direct medical costs related to the drugs varied for the different strategies, depending on the specific drugs and treatment duration (Supporting Table S1).

In Vietnam, the drug cost for a 12-week treatment course with sofosbuvir/daclatasvir (the most widely used DAA regimen^4^) in 2023 was US$963 (US$80.25 per week).^7^ This was used as the base-case scenario, since the trial also showed non-inferiority between this and sofosbuvir/velpatasvir (5% non-inferiority margin). As DAA costs vary widely between countries,^7^ we considered seven alternative scenarios for the cost of 12-weeks of DAAs. The cost of shorter DAA exposure was derived from these values (accounting for the relative duration of treatment). The cost of Peg-IFN (Peginterferon alfa-2a) was assumed to be US$84.20 per week (based on procurement within VIETNARMS). Following HTD records, ribavirin was assumed to cost US$0.86 per day. In the base-case, we assumed the DAA costs for retreatment were the same as for first-line treatment (as costs for sofosbuvir/daclatasvir and sofosbuvir/velpatasvir are similar in Vietnam^4^), with variation on the relative cost explored in sensitivity analysis.

Based on information from Vietnam's Ministry of Health,^20^ we assumed that Vietnam's national health insurance program would cover 50%, 30% and 80% of the costs of DAAs, Peg-IFN and ribavirin.

#### Direct medical costs—medical services and monitoring

The direct medical costs related to medical services (e.g. medical tests, consultation fees) were based on information about the type of laboratory tests and when they were performed, following Vietnam's 2021 treatment guidelines for hepatitis C^15^ (Supporting Table S2). Note that this could be different from what occurred in the trial itself. The corresponding unit costs for the different services were based on the price list of healthcare services used at HTD in 2021 (Supporting Table S3). We assumed that Vietnam's national health insurance program would cover 80% of these costs.^21^

#### Direct non-medical costs and productivity costs

Data on the direct non-medical and productivity costs incurred by patients when accessing treatment at the HTD outpatient clinic were collected concurrently with the trial. Based on this, it was assumed that patients would incur a mean of US$25.57 (2023 prices) in direct non-medical and productivity costs per visit, including the costs incurred by their informal caregivers.^22^ Productivity costs were calculated in line with the human capital approach (using Gross Domestic Product (GDP) per capita to value productivity losses (US$14.60 per day).^22^ Costs were inflated from 2021 to 2023 values using Vietnam's GDP deflators.^19^

It was assumed that for a standard 12-week DAA treatment course, three clinic visits would be needed. The number and timing of the visits for the alternative treatment strategies varied according to their respective resource requirements (outlined in Supporting Table S2).

### Output and sensitivity analysis

The primary output of the analysis was the cost per case cured. For each alternative strategy, we also calculated the difference in the cost per cure versus the standard 12-week DAA treatment and further disaggregated these differences by the source of expenditure—distinguishing costs borne by healthcare providers from those paid by patients. In addition, the corresponding minimum “breakpoint” DAA drug cost value for a standard 12-week course, which would result in an alternative strategy having a lower total cost per cure, was also calculated.

The final results incorporate the uncertainty in the relative effectiveness of each strategy in achieving SVR compared to standard treatment by rerunning the cost model 10,000 times under assumptions for effectiveness drawn from a beta distribution that matches the main trial results^13^ (Supporting Table S1). We also performed a scenario analysis for the costs, as outlined in Table 1.

**Table 1:** Scenario analysis for the costs.

Parameter	Default assumptions (base-case)	Range investigated in the scenario analysis
Cost of DAAs	US$80.25 per week (US$963 for a 12-week standard course of sofosbuvir/daclatasvir)	US$50–1500 per 12-week standard course^7^
The relative DAA drug cost of the regimen used during a retreatment	The 1st line and retreatment regimens have the same DAA cost (varied as outlined above)	200% increase in the DAA drug cost for the retreatment regimen
HCV-RNA viral load test cost	US$59.36	US$23.82–121.78^a^^,^^23^
Cost of Peg-IFN	US$84.20 per week	US$52.34–172.37 per week^a^^,^^24^^,^^25^
The costs for patients accessing the treatment (direct non-medical and productivity costs)	US$25.57 per visit	US$11.59–37.04 per visit The lower and higher cost scenarios were based on whether the patient lives in the same city as the treatment center or needs to travel from another province.^22^
Number of clinic visits	See Supporting Table S2	One additional visit for the three alternative strategies relative to standard treatment from what was assumed for the base-case
Health insurance program contribution	50% of the cost of DAAs 80% of the cost of monitoring/tests 30% of the cost of Peg-IFN 80% of the cost of ribavirin medicines	100% of all direct medical costs
Additional lost productivity due to adverse events.	Not captured	For those taking Peg-IFN + DAA, 39% lose 2 days of work due to flu-like symptoms^13^ (valued based on the GDP per capita)
Non-drug costs	Varied separately as outlined above	−50% to +100%

a Adjusted to US$ 2023 costs using^26^ US inflation rates.

### Ethics

Ethical approval for the VIETNARMS trial was obtained from local hospital ethics committees at the Hospital for Tropical Diseases and the National Hospital of Tropical Disease; the Ministry of Health, Viet Nam; Imperial College London, UK; and the University of Oxford, UK (ref 110-0319/35CN).

### Role of the funding source

The funder of the study had no role in the study design, data collection, data analysis, data interpretation, or writing of the report.

## Results

### Unit costs of the treatment regimens

The estimated mean unit costs of the different treatment regimens are summarized in Table 2 and Supporting Figure S2, assuming a base-case drug cost of US$963 for 12-weeks of DAAs. Note that the unit costs only consider the cost of that strategy itself and do not consider the proportion needing retreatment (which is captured in the cost per cure). The total mean cost of standard treatment was US$1291. Most comprised of drug costs (75%), followed by the direct medical costs associated with monitoring/tests (19%). Of this total, 53% was incurred by the healthcare provider and 47% by the patients.

**Table 2:** Projected mean unit cost (US$) of the different regimes assuming a base-case drug cost of US$963 for 12-weeks of DAAs.

Cost type	Standard treatment	Response-guided			Induction-maintenance	Peg-IFN + DAA	Retreatment
		4-week duration	8-week duration	12-week duration			
Direct medical costs–Medical services and monitoring	251.55	310.91	312.65	312.65	251.55	261.98	166.68
Direct medical costs–Drug costs	963.00	321.00	642.00	963.00	733.71	657.80	1035.54
Direct non-medical costs and Productivity costs	76.70	76.70	102.26	102.26	76.70	153.39	76.70
Total paid by patient	608.50	299.38	485.79	646.29	493.86	602.05	606.04
Total paid by healthcare provider	682.74	409.23	571.12	731.62	568.09	471.12	672.88
**Total (Overall)**	1291.24	708.61	1056.91	1377.91	1061.96	1073.17	1278.92

Costs are in 2023 US$ costs.

Note that the unit costs reflect only the cost of delivering each strategy itself; they do not incorporate the proportion of individuals requiring retreatment, which is instead accounted for in the cost per cure.

The costs for patients accessing the treatment (direct non-medical and productivity costs) varied modestly across treatment regimens, with mean values ranging from US$77 to US$153, with higher costs for Peg-IFN + DAA, reflecting the number of treatment visits for injections.

The costs of the response-guided strategy differed among the three strata receiving different DAA durations based on 1-week viral load. The direct medical costs associated with monitoring were higher for the response-guided strategy, as they required an additional viral load test. The unit costs for those receiving 4- and 8-week duration of DAAs were lower than the standard 12 week DAA treatment, as fewer drugs were used. In contrast, the unit cost for the response-guided strata receiving 12-week duration of DAAs was higher than the standard treatment, as there were no drug-related savings but additional monitoring/test-related costs.

The unit cost of induction-maintenance was lower than the standard 12 weeks due to savings in drug-related costs; the costs for other categories remained the same as the standard treatment.

The unit cost of Peg-IFN + DAA was also lower than standard treatment. For the default assumptions, the cost savings associated with reduced DAA exposure outweighed the additional cost of adding Peg-IFN. The proportion of costs paid by the patient was higher for this strategy because of the lower contribution of the health insurance program to Peg-IFN and the additional treatment visits required (Table 2).

Supporting Table S4 presents projected unit costs under varying DAA price scenarios, highlighting how total and patient-incurred costs differ across treatment strategies. While total costs increase across all regimens as DAA prices rise, the rate and magnitude of increase vary by strategy.

### Projected cost per cure of the different strategies

**Fig. 1:**
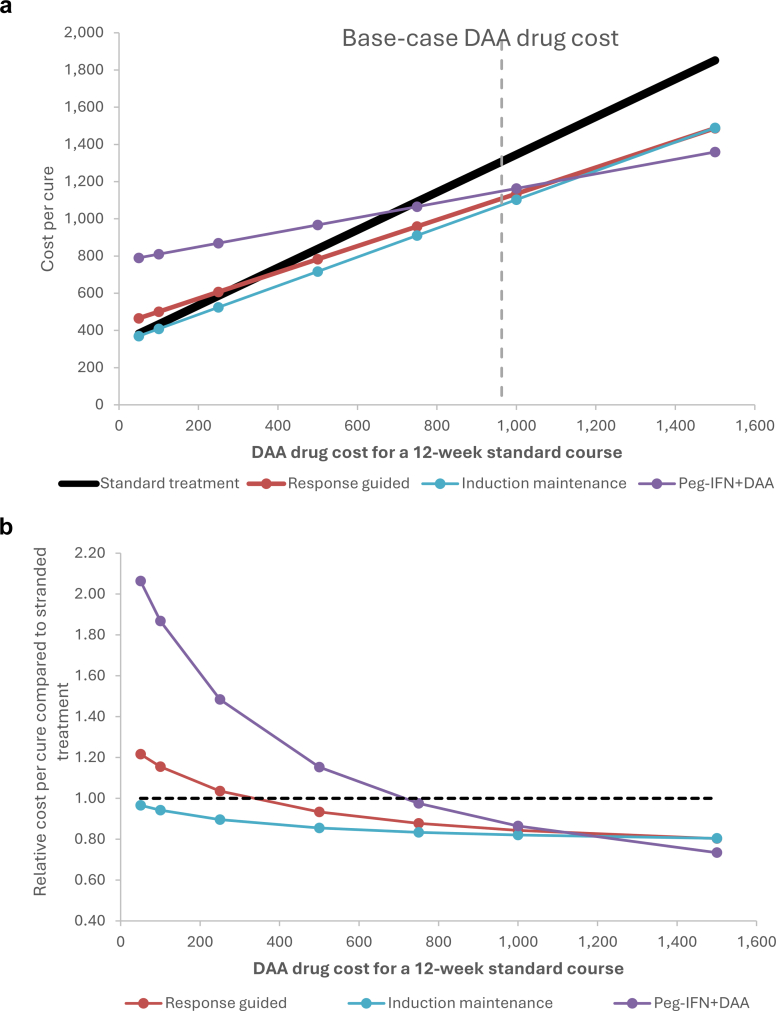
**Projected (a) mean cost per cure for the different treatment strategies and (b) relative mean cost per cure of alternative strategies compared to the stranded treatment.** Panel (a) plots cost per cure against the DAA drug cost for a 12-week course, with a dotted vertical line indicating the baseline cost of US$963. Panel (b) shows the cost per cure of each alternative relative to the standard treatment, with the dotted horizontal line at 1.0 marking the threshold for cost savings. All costs are expressed in 2023 US$ costs.

**Fig. 2:**
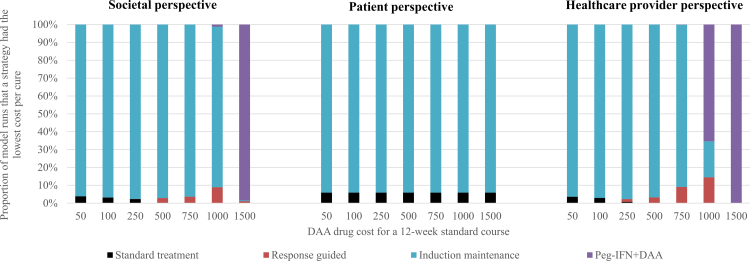
**The proportion of model simulations in which each strategy had the lowest projected cost per cure under varying DAA cost scenarios.** The model was run for 10,000 simulations.

Each of the three strategies had a lower mean cost per cure than the standard 12-week DAA treatment (Fig. 1 and Supporting Table S5). At the base-case DAA drug cost, the savings were very similar but marginally greater for induction-maintenance. The relative cost per cure was notably influenced by the assumed cost of the DAAs (Fig. 2). For the default assumptions, the cost per cure was consistently lower for induction-maintenance. When DAA costs were lower, induction-maintenance had the lowest relative cost, followed by the response-guided strategy. With higher DAA costs, the savings from Peg-IFN became more pronounced. The estimated mean breakpoint DAA drug cost for a 12-week course that would result in a lower cost per cure with an alternative strategy than the current standard strategy was US$318 and US$703 for response-guided and Peg-IFN + DAA strategies, respectively. Within the uncertainty analysis, the corresponding breakpoint values that would result in 95% of the model runs projecting cost-savings were US$410 and US$812, respectively.

**Fig. 3:**
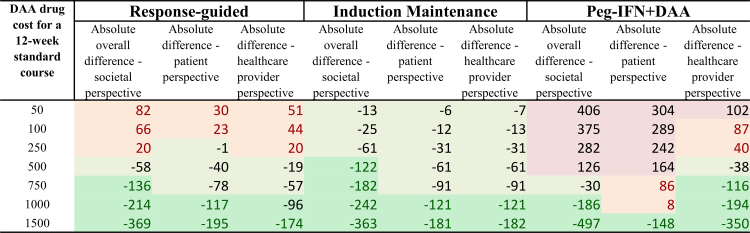
**Difference in the estimated mean cost per cure relative to the standard treatment stratified by different perspectives.** The color shading illustrates both the size and direction of cost differences in mean cost per cure compared to the standard treatment. Dark green indicates cost savings of US$100 or more, while light green represents smaller savings between US$1–99. Orange signals a moderate increase in cost within the US$1–99 range, and red marks a more substantial increase of US$100 or more. Costs are in 2023 US$ costs.

Fig. 2 displays the proportion of model simulations in which each alternative strategy achieved the lowest cost per cure across a range of DAA drug costs, analyzed from societal, patient, and healthcare provider perspectives. At lower drug costs, induction-maintenance frequently emerged as the most cost-efficient from all perspectives. The standard 12-week DAA course was rarely the optimal choice under any scenario. As DAA costs increased, the optimum strategy differed across the perspectives, with Peg-IFN + DAA becoming increasingly dominant from the societal and healthcare provider perspectives. In contrast from the patient's perspective, the response-guided strategy emerged as the most favorable. This difference is because the cost of each strategy falls on the healthcare provider and the patient differently (Fig. 3). Of note, with the default assumptions (reflecting Vietnam's co-payments rates), Peg-IFN + DAA could have a lower cost per cure than the standard treatment, saving costs for the healthcare provider relative to the current treatment strategy, while imposing additional costs on the patients (Fig. 3).

The differences in the cost per cure have been presented on a per-patient bias. If these were applied to a larger programmatic context, such as targeting all hepatitis C patients in Vietnam, these savings, though small in relative terms, would become more substantial on an absolute basis (Supporting Table S6).

### Scenario analysis

We performed a sensitivity analysis for several key parameters (Supporting Tables S7–S10). Increasing the relative DAA drug cost for the retreatment regimen had little impact on the outcomes, slightly increasing the projected cost per cure (Supporting Table S9).

Varying the assumed unit cost of the viral load test between US$23.82–121.78 had little impact on the projected cost per cure of the different strategies (Supporting Table S9). However, it did influence the breakpoint DAA drug cost, resulting in the response-guided strategy becoming cost-saving (Supporting Table S7). When using the lower cost range for this test, the response-guided strategy was more likely to have the lowest cost per cure (Supporting Table S8).

Varying the cost of Peg-IFN had a notable impact on the cost per cure of Peg-IFN + DAA and the breakpoint DAA drug cost, for which it became cost-saving (Supporting Figure S3, Supporting Tables S7 and S9).

The assumed non-medical and productivity costs incurred by patients to access treatment had a relatively small impact on the total projected cost per cure (Supporting Table S9). (Supporting Table S10). These costs had the greatest influence on Peg-IFN + DAA, as it required more treatment visits compared to standard treatment. This impact was exacerbated when using non-medical/productivity cost values related to patients living outside the city where the treatment centre was located (Supporting Table S10). This would make the Peg-IFN + DAA strategy less likely to be optimum in settings with centralized treatment.

When assuming that each of the three strategies required one additional visit from the base-case assumptions compared to the standard treatment, the projected cost per cure was not notably affected (Supporting Table S7). However, it decreased the possibility of alternative strategies generating cost savings at very low DAA costs (Supporting Tables S7 and S9). This was particularly pertinent for induction-maintenance.

The assumed health insurance co-payment contribution did not affect the projected cost per cure but influenced the distribution of costs and savings (Supporting Table S10). Assuming that the health insurance program would cover all direct medical costs, it would shift much of the cost (and potential savings) to the healthcare provider. However, patients still incur costs when accessing treatment.

Including additional lost productivity due to potential adverse events for the Peg-IFN + DAA strategy also affected its cost per cure and the projected cost breakpoint, but the impact was smaller than that observed for assumptions related to the cost of Peg-IFN or the costs incurred by patients when accessing treatment.

Varying all the non-drug cost simultaneously influenced the projected cost per cure across all strategies, but these changes within the range investigated did not substantially alter the relative ranking of strategies or the conditions under which they became cost-saving. A lower non-drug cost scaling factor benefited the response-guided strategy the most (due to the impact on the viral load test cost).

## Discussion

Alternative strategies that reduce the amount of DAA exposure could reduce the costs associated with hepatitis C treatment for the health sector and for patients. Indeed, under current assumptions for costs, each of the three alternative treatment strategies investigated in the VIETNARMS trial (response-guided, induction-maintenance, and Peg-IFN + DAA) were projected to have a lower cost per cure and hence the potential to generate cost-savings, and the savings were similar across the alternative strategies. However, this potential for cost savings and which strategy had the lowest cost per cure were highly dependent on the assumed cost of DAAs and other treatment-related costs which are variable, and likely to change over time. This highlights that policy recommendations should adapt to the local context and the importance of not using a one-size-fits-all approach.

When considering alternative DAA strategies, it is important to consider the balance of the costs incurred by healthcare providers versus patients. This will be highly sensitive to the local context and the assumed insurance co-payment rates for the specific drugs and services in question. These rates will vary across different countries and require further investigation in a range of settings. Alternative strategies that require more visits to health facilities will increase the direct non-medical/productivity costs incurred by patients. In settings where treatment is centralized, this extra burden will be disproportionately larger for patients who do not live in the same area as the main health facility. This was most pertinent to Peg-IFN + DAA, which required six outpatient clinic visits (four for weekly injections). Note this could change if treatment was self-administered and this should be evaluated. These factors are relevant for policymakers to consider, as even if a new treatment strategy is cheaper overall, it may still be more expensive for patients. If ignored, it has the potential to generate inequity and access problems. It is also important to note that there are inherent benefits of shorter treatment durations that go beyond cost savings. A four-week co-treatment with Peg-IFN could be used to increase coverage for certain hard-to-reach population groups, even if it has a higher cost per cure compared to standard treatment. This highlights the importance of not using a one-size-fits-all approach and tailoring strategies to the local context of each country.

Regarding the uncertainty around non-inferiority effectiveness outcomes of the different strategies, it is important to treat the point effectiveness estimates with a degree of caution. The analysis of the trial data found that there was a 26% probability that induction–maintenance was inferior to standard treatment, a 99% probability that Peg-IFN + DAA was inferior to standard treatment, and a 100% probability that response-guided was inferior to standard treatment.^13^ Due to this, we used the distribution of effectiveness estimates and not point estimates when projecting the cost per cure.

Although the focus of this study was Vietnam (which is a high cost outlier compared to many other LMICs^7^), these results are also pertinent to high-income settings, where DAAs are still typically expensive, highlighting the need for further evaluation of these alternative strategies in other settings. It does not follow that these options should necessarily replace standard treatment, but it does highlight the utility of these alternative strategies in certain situations/patient groups. It is also important to consider the results in light of any changes to hepatitis C treatment that may occur, such as the availability of lower-cost RNA tests or the potential use of an 8-week treatment strategy as standard (although not recommended for sofosbuvir-based combinations in WHO guidelines^27^). The points at which cost savings emerge for the different strategies were sensitive to different parameters. For example, the breakpoint for the response-guided strategy was sensitive to the cost of the viral load test. In contrast, the breakpoint for induction-maintenance was sensitive to the number of treatment visits, and the breakpoint for Peg-IFN + DAA was sensitive to the cost of Peg-IFN.

This study adds to the growing evidence base of economic evaluations investigating shorter DAA treatments for hepatitis C.^28^^,^^29^ Notably, Morgan et al.^28^ modeled the population-level impact of introducing 8-week DAA treatment courses under budget constraints, while Fawsitt et al.^29^ evaluated the cost-effectiveness of an 8-week regimen in genotype 1, non-cirrhotic, treatment-naïve patients using UK-based data. A key distinction of our analysis is that it evaluates a response-guided treatment strategy, rather than uniformly reducing treatment duration for all patients, and it draws on trial data from a non-high-income country context.

One important limitation is that the effectiveness data are derived from a trial population that had a high proportion of genotype-6 infections, consisted of adults with minimal-to-moderate fibrosis, and was restricted to hospital-based participants in Vietnam.^13^ Furthermore, within our projections of the cost per cure, we did not account for the small number of patients who would potentially fail retreatment and may not eventually be cured at all. Within the trial this only occurred for 2 (0.003%) out of the 609 participants.^13^ In addition, we did not formally differentiate between specific DAA drug regimens used for initial and retreatment. However, we found that varying the relative cost of the DAA regimen used for retreatment had little impact. Moreover, the use of a cost-minimization framework meant that any small differences in health effects between treatment strategies were not accounted for. We also note that we did not fully account for the cost or loss of quality of life associated with treatment-related adverse effects, which can particularly affect the benefit of the Peg-IFN + DAA strategy (which had the majority of the reported adverse reactions within the trial^13^). Nevertheless, severe adverse reactions were rare across all strategies investigated.^13^

The efficacy of the alternative strategies was based on a trial conducted in Vietnam.^13^ For each strategy, we used the distribution of the aggregate effectiveness values and did not stratify by genotype or specific DAA drug used, given there was no evidence of heterogeneity by these, or other, factors in the trial. Additionally, the costs used in the analysis were parameterized based on the Vietnamese context. It should be noted that differences in clinical practice could influence costs in other countries. Furthermore, the health insurance contributions assumed for the results with the default assumptions were based on the typical values experienced by patients in Vietnam. It should be noted that there is some variation in these values, and certain vulnerable populations have higher co-payment rates for certain medical expenses. In contrast, patients who are uninsured or do not follow the designated referral pathway would receive no health insurance contribution.

Treatment completion in routine care will likely be lower than in the trial because patients face logistical, financial, and social barriers that trials mitigate. Shorter regimens with lower pill burden would be expected to achieve higher real-world completion rates. Peg IFN + DAA strategy poses additional adherence/acceptability challenges because Peg-IFN is injectable and associated with more side effects; however, the shorter 4-week course may partially mitigate these risks by reducing the window for dropout and cumulative side effect burden. It could also have higher compliance for particularly hard-to-reach population groups. Further studies are needed to consider the acceptability of the strategies to patients.

The alternative strategies investigated in the VIETNARMS trial have the potential to have a lower cost per cure compared to the standard 12-week DAA treatment. However, although these alternative strategies are promising, recommendations should be adapted to the local context.

## Contributors

HCT and TBH conceived and designed the study. HCT led the analysis. SA and HAN supported the analysis. HCT drafted the manuscript. LMH, JVN, DTT, NTD, GET, ASW, NVVC, GSC and TBH contributed to the interpretation of results and critically revised the manuscript for intellectual content. All authors read and approved the final manuscript, had full access to all the data, and accept responsibility for the decision to submit the manuscript for publication. HCT and TBH accessed and verified the underlying data. HCT is the guarantor.

## Data sharing statement

All data generated or analyzed during this study are presented in the manuscript and its supplementary files. The calculation model is available from the corresponding author upon reasonable request.

## Declaration of interests

The authors declare that they have no competing interests.

## Appendix A Supplementary data

Supporting Information 2

SEARCH investigators

Supplementary file captions
