# Supplementary material for: Economic evaluation of alternative hepatitis C treatment options: a post hoc analysis of the VIETNARMS trial
Source: eClinicalMedicine. 2026 May 7;95:103969. doi: 10.1016/j.eclinm.2026.103969 (PMC13156567; doi:10.1016/j.eclinm.2026.103969)
Supplement: Supporting Information 1 [file mmc1.pdf]

Supporting information: Economic evaluation of alternative Hepatitis C treatment options: Analysis of the VIETNARMS trial

Hugo C Turner[1*], Sayara Ahmed[1], Huyen Anh Nguyen[2], Le Manh Hung[3], Jennifer Van Nuil[2,4], Thuan Dang Trong[2], SEARCH investigators[5], Nguyen Thanh Dung[3], Guy E Thwaites[2,4], Ann Sarah Walker[6], Nguyen Van Vinh Chau[3], Graham S Cooke[7] and Timothy B. Hallett[1]

[1] MRC Centre for Global Infectious Disease Analysis, School of Public Health, Imperial College London, Norfolk Place, London, United Kingdom

[2] Oxford University Clinical Research Unit, Wellcome Trust Major Overseas Programme, Ho Chi Minh City, Vietnam.

[3] Hospital for Tropical Diseases, Ho Chi Minh City, Vietnam

[4] Centre for Tropical Medicine and Global Health, Nuffield Department of Medicine, University of Oxford, Oxford, United Kingdom

[5] The members of the SEARCH investigators affiliation are listed in the Acknowledgements

[6] MRC Clinical Trial Unit at UCL, University College London, United Kingdom

[7] Department of Infectious Disease, Imperial College London, United Kingdom

* Corresponding Author hugo.turner@imperial.ac.uk

Contents

Further methodological details ... 3

Supporting Figure S1: In-country drug prices for a 12-week course of DAA treatment ... 4

Supporting Table S1: Overview of DAA exposure and effectiveness for the different treatment regimens ... 5

Supporting Table S2: Number of tests required for the different strategies investigated ... 6

Supporting Table S3: Assumed unit costs ... 7

Supporting Figure S2: Projected unit costs of the different treatment regimens investigated, stratified by a) cost type and b) payer type ... 8

Supporting Table S4: Projected unit cost (and cost incurred by the patients) of the different treatment regimens for different scenarios regarding the cost of DAAs ... 9

Supporting Table S5: Projected cost per cure of the different strategies under varying DAA cost scenarios ... 10

Supporting Table S6: Hypothetical absolute difference in the projected cost to cure all Hepatitis C infections in Vietnam relative to standard treatment, stratified by perspective ... 11

Supporting Table S7: The projected mean breakpoint drug cost for 12-weeks' standard treatment resulting in the alternative strategy being cost-saving under the different cost scenarios ... 12

Supporting Table S8: The projected strategy with the lowest cost per cure for different DAA drug costs under the cost scenarios ... 13

Supporting Table S9: Mean cost per cure across the different cost scenario analyses ... 14

Supporting Table S10: Difference in the mean cost per cure relative to the standard treatment stratified by different perspectives across the scenario analysis ... 17

Supporting Figure S3: Scenario analysis regarding the weekly cost of Peg-IFN ... 22

Further methodological details

More general data and information regarding the characteristics/demographics of these study populations are available within the main trail paper VIETNARMS trial, and from the General Statistics Office of Vietnam [1].

No specific health economic analysis plan was developed or previously published for this study. In addition, there were no approaches to engage patients, the general public, or stakeholders in the design of this economic evaluation.

In terms of characterising heterogeneity and distributional effects, it was assumed that the benefit of the intervention was equally experienced among the targeted populations. No specific adjustments were made to reflect priority populations. However, the impact within the different settings was investigated. In addition, the lower and higher scenarios for the non-medical and productivity costs were based on whether the patient lives in the same city as the treatment centre or needs to travel from another province

Data on direct non-medical and productivity costs incurred when accessing treatment at the Hospital for Tropical Diseases (HTD) outpatient clinic were collected concurrently with the trial from 102 participants [2]. Just under half lived in Ho Chi Minh City (45%), with the remainder residing in other southern provinces. The mean monthly household income was US$678, though this concealed substantial differences: households in Ho Chi Minh City earned almost twice as much as those outside the city. Travel patterns differed markedly by residence. Participants living in Ho Chi Minh City primarily used motorbikes, whereas those from outside the city typically travelled by bus. The average travel time was nearly one hour for Ho Chi Minh City residents and almost five hours for those living elsewhere. Consequently, the total time spent per clinic visit averaged approximately half a day for Ho Chi Minh City residents and a full day for those from outside Ho Chi Minh City. Overall, 39% of participants were accompanied by an informal caregiver. Among these, 30% were from Ho Chi Minh City and 70% from other southern provinces.

a) Sofosbuvir/daclatasvir regimen

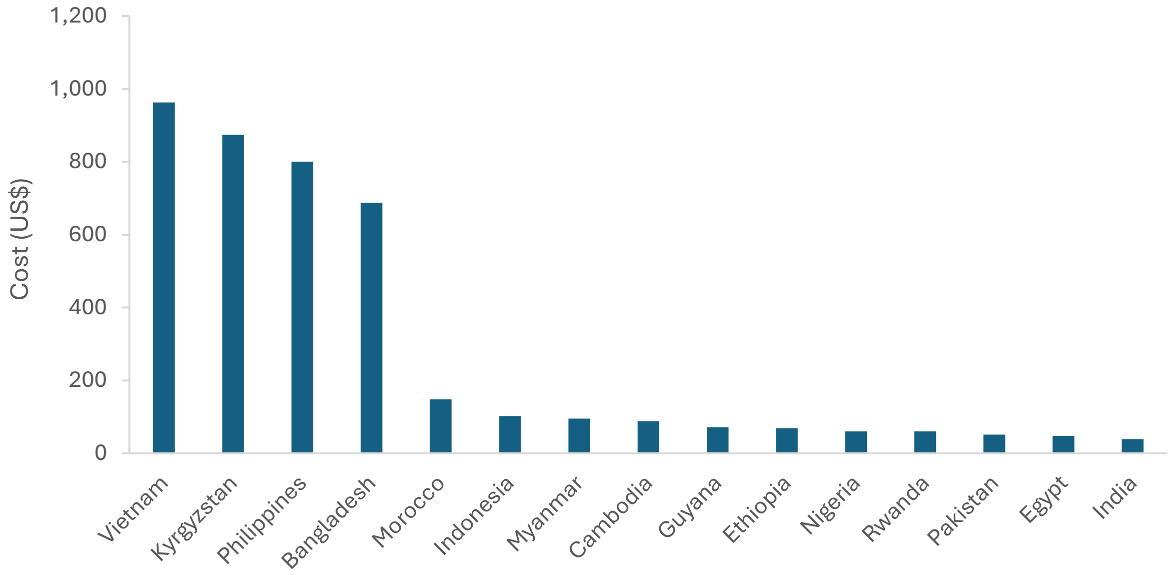

b) Sofosbuvir-velpatasvir regimen

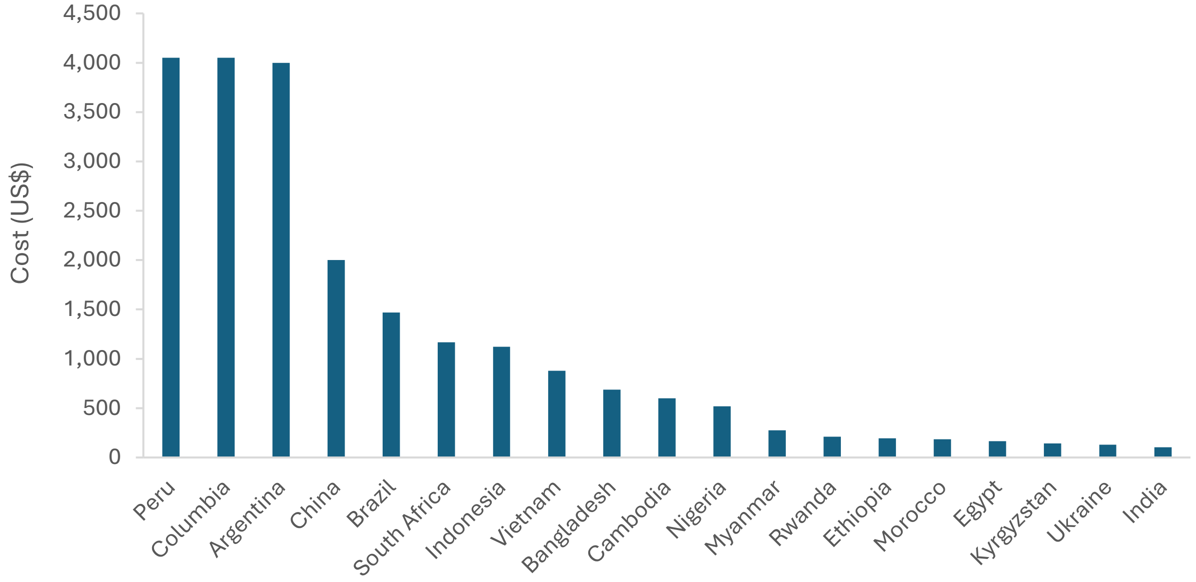

Supporting Figure S1: In-country drug prices for a 12-week course of DAA treatment. *Adapted from [3].*

Supporting Table S1: Overview of DAA exposure and effectiveness for the different treatment regimens

Treatment regimen	Treatment efficacy	Difference in effectiveness relative to standard treatment (90% Crls)	Drug frequency		
			DAA	Peg-IFN	Ribavirin
Standard treatment	98.7% [4]	-	Once a day for 12 weeks	-	-
Response-guided – 4-week duration (25%[a])			Once a day for 4 weeks	-	-
Response-guided – 8-week duration (60%[a])	Overall: 94.1%	Overall: -5.7% (-9.6%, -2.3%) [4]	Once a day for 8 weeks	-	-
Response-guided – 12-week duration (15%[a])			Once a day for 12 weeks	-	-
Induction-maintenance	98.7%	+0.6% (-1.1%, 2.7%) [4]	Once a day for 2 weeks followed by every weekday for 10 weeks	-	
Peg-IFN+DAA	94.1%	-4.5% (-8.3%, -1.3%) [4]	Once a day for 4 weeks	Once a week for 4 weeks	-
Retreatment	100% (assumed)	-	Once a day for 12 weeks	-	4 tablets a day for 12 weeks

[a] The numbers represent the proportion receiving the different durations of the response-guided therapy strategy based on their week-1 viral load result.
Crls: Credible intervals

Supporting Table S2: Number of tests required for the different strategies investigated

Test type	Test	Standard treatment	Re-treatment	Response-guided (4-week duration strata)	Response-guided (8-week duration strata)	Response-guided (12-week duration strata)	Induction maintenance	Peg-IFN+DAA
Ultrasound	Abdominal ultrasound	2	2	2	2	2	2	2
	Fibro-scan	2	2	2	2	2	2	2
Blood tests	Full blood count	2	3	2	2	2	2	2
	Prothrombin	2	3	2	2	2	2	2
	Blood sugar and fats	1	1	1	1	1	1	1
	Creatinine	2	3	2	2	2	2	2
	Liver function tests	2	2	2	2	2	2	2
Immunoassays	Human immunodeficiency virus	1	0	1	1	1	1	1
	Hepatitis B surface antigen	1	0	1	1	1	1	1
	Alpha-fetoprotein (AFP)	2	1	2	2	2	2	2
	Thyroid-stimulating Hormone	0	0	0	0	0	0	2
Molecular biology tests	HCV-RNA viral load test	2	2	3	3	3	2	2
	HCV genotype real-time PCR	1	0	1	1	1	1	1
Clinical consultations	Number of examinations/visits[a]	3	3	3	4	4	3	6

Values based on what is expected to occur in terms of the practical implementation of these strategies, rather than what was observed in the trial.

[a] It was assumed that the standard 12-week treatment course would require three clinic visits: one at treatment initiation (day 0), one during treatment (approximately day 28), and one follow-up visit 12 weeks after treatment completion. For the induction–maintenance strategy, the same number of clinic visits was assumed, with the mid-treatment visit shifted earlier to day 14. The response-guided strategy was assumed to require three visits for the 4-week stratum, with the mid-treatment assessment moved to day 7, and four visits for the 8- and 12-week strata, adding an extra visit to accommodate the day 7 test. The Peg-IFN+DAA strategy was assumed to require six visits, accounting for the four Peg-IFN injections administered during treatment alongside the treatment initiation and follow-up visits.

Supporting Table S3: Assumed unit costs

Item	Unit cost (VND)	Unit cost (US$)
Drugs		
DAA	Varied	Varied
Peg-IFN	1,950,000 per week	US$84.20 per week
Ribavirin	20,000 per day (4,000 per tablet)	US$0.86 per day
Service/tests		
Abdominal ultrasound	46,821	1.97
Fibro-scan	87,777	3.69
Full blood count	49,274	2.07
Prothrombin	67,726	2.85
Blood sugar and fats	51,621	2.17
Creatinine (blood)	22,931	0.96
Liver function tests	89,270	3.75
Human immunodeficiency virus	113,054	4.75
Hepatitis B surface antigen	279,755	11.76
Thyroid-stimulating Hormone	62,180	2.61
Alpha-fetoprotein	97,696	4.11
HCV-RNA viral load test	1,412,107	59.36
HCV genotype real-time PCR	1,668,077	70.13
Clinical consultation fees	41,275	1.74

a)

b)

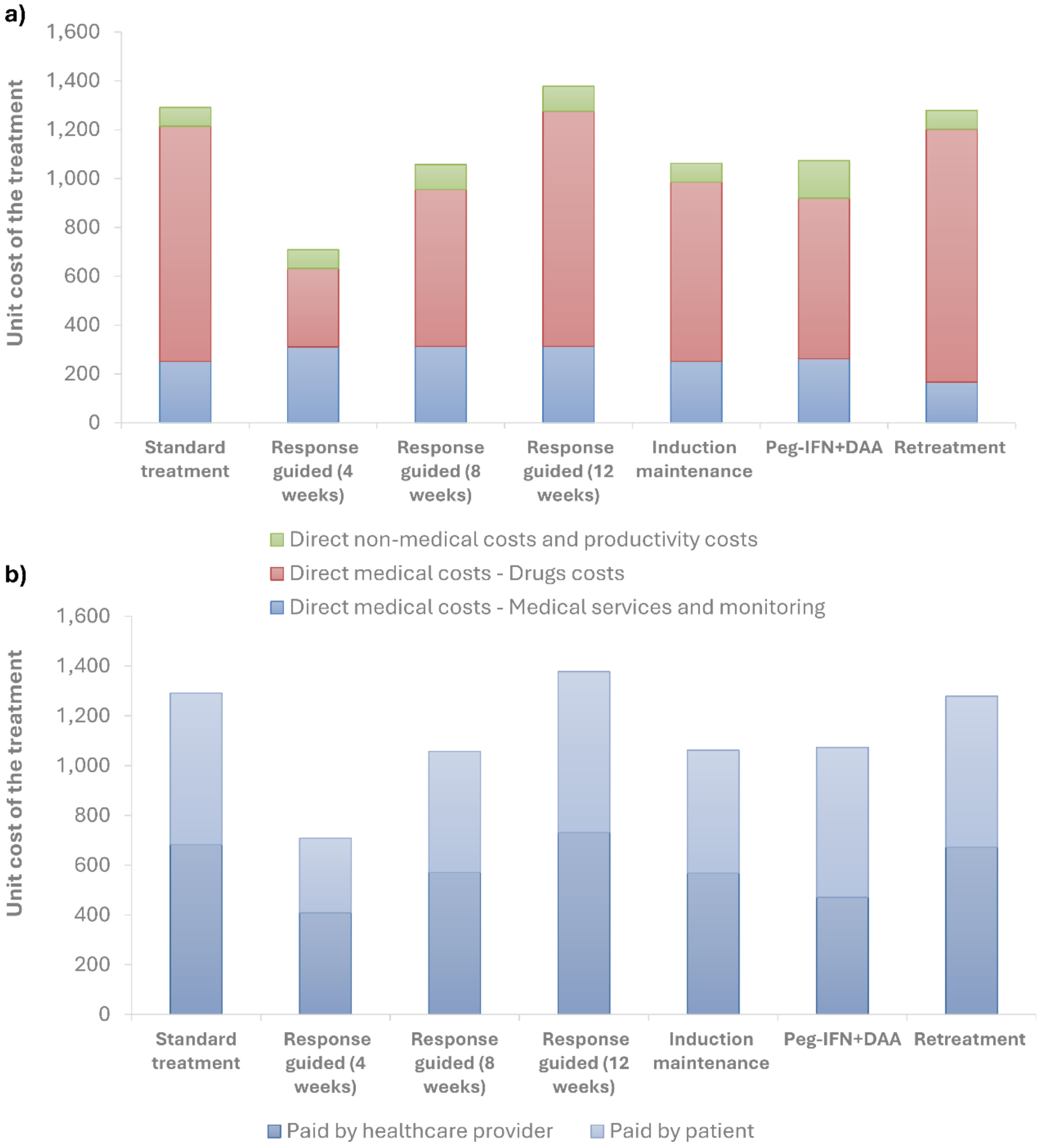

Supporting Figure S2: Projected unit costs of the different treatment regimens investigated, stratified by a) cost type and b) payer type. *The results assumed a DAA drug cost of US$963 for a standard 12-week course of DAA. Costs are in 2023 US$ costs*

Supporting Table S4: Projected unit cost (and cost incurred by the patients) of the different treatment regimens for different scenarios regarding the cost of DAAs.

Drug cost for a 12-week standard course of DAAs	Standard treatment	Response-guided			Induction maintenance	Peg-IFN+DAA	Retreatment
		4-week duration	8-week duration	12-week duration			
**50**	378.24 (152.00)	404.27 (147.21)	448.24 (181.46)	464.91 (189.79)	366.34 (146.05)	768.84 (449.88)	365.92 (149.54)
**100**	428.24 (177.00)	420.94 (155.54)	481.57 (198.12)	514.91 (214.79)	404.43 (165.10)	785.50 (458.21)	415.92 (174.54)
**250**	578.24 (252.00)	470.94 (180.54)	581.57 (248.12)	664.91 (289.79)	518.72 (222.24)	835.50 (483.21)	565.92 (249.54)
**500**	828.24 (377.00)	554.27 (222.21)	748.24 (331.46)	914.91 (414.79)	709.19 (317.48)	918.84 (524.88)	815.92 (374.54)
**750**	1078.24 (502.00)	637.61 (263.88)	914.91 (414.79)	1164.91 (539.79)	899.67 (412.72)	1002.17 (566.55)	1065.92 (499.54)
**1000**	1328.24 (627.00)	720.94 (305.54)	1081.57 (498.12)	1414.91 (664.79)	1090.15 (507.96)	1085.50 (608.21)	1315.92 (624.54)
**1500**	1828.24 (877.00)	887.61 (388.88)	1414.91 (664.79)	1914.91 (914.79)	1471.10 (698.43)	1252.17 (691.55)	1815.92 (874.54)

The values incurred by the patients are denoted in the brackets. Costs are in 2023 US$ costs.

Supporting Table S5: Projected cost per cure of the different strategies under varying DAA cost scenarios

	Mean cost per cure (standard deviation)			
**DAA drug cost for a 12-week standard course**	**Standard treatment**	**Response-guided**	**Induction maintenance**	**Peg-IFN+DAA**
**Base-case DAA cost**	1308	1106 (29)	1074 (76)	1145 (28)
**50**	383	465 (8)	370 (22)	789 (8)
**100**	434	500 (9)	408 (25)	809 (9)
**250**	586	606 (13)	524 (34)	867 (12)
**500**	839	781 (19)	717 (48)	965 (18)
**750**	1092	957 (24)	910 (63)	1062 (23)
**1000**	1345	1132 (30)	1103 (78)	1160 (28)
**1500**	1852	1483 (41)	1489 (108)	1355 (39)
**The breakpoint drug cost for a 12-week standard treatment resulting in the alternative strategy being cost-saving**		318 (410)[1]	Cost saving[2] (-4)[1]	703 (812)[1]

[1] *Value in brackets represents the breakpoint value that would result in 95% of the model runs projecting cost-savings.*
[2] *As the strategy is on average generating cost saving it does not have a corresponding breakpoint.*
Costs are in 2023 US$ costs.

Supporting Table S6: Hypothetical absolute difference in the projected cost to cure all Hepatitis C infections in Vietnam relative to using standard treatment, stratified by perspective

	Response guided			Induction maintenance			Peg-IFN+DAA		
**DAA drug cost for a 12-week standard course**	Absolute overall difference - societal perspective	Absolute difference - patient perspective	Absolute difference - healthcare provider perspective	Absolute overall difference - societal perspective	Absolute difference - patient perspective	Absolute difference - healthcare provider perspective	Absolute overall difference - societal perspective	Absolute difference - patient perspective	Absolute difference - healthcare provider perspective
50	75,342,799	28,033,856	47,308,943	-12,009,187	-5,908,295	-6,100,892	374,014,623	280,067,224	93,947,399
100	61,033,667	20,879,290	40,154,377	-23,108,059	-11,457,731	-11,650,328	345,334,432	265,727,128	79,607,304
250	18,106,270	-584,409	18,690,679	-56,404,673	-28,106,038	-28,298,635	259,293,857	222,706,841	36,587,016
500	-53,439,391	-36,357,239	-17,082,152	-111,899,030	-55,853,217	-56,045,813	115,892,899	151,006,362	-35,113,463
750	-124,985,052	-72,130,070	-52,854,982	-167,393,387	-83,600,395	-83,792,992	-27,508,060	79,305,882	-106,813,942
1000	-196,530,714	-107,902,901	-88,627,813	-222,887,744	-111,347,574	-111,540,170	-170,909,018	7,605,403	-178,514,421
1500	-339,622,036	-179,448,562	-160,173,474	-333,876,458	-166,841,931	-167,034,527	-457,710,934	-135,795,555	-321,915,380

Calculations are based on a projected total of 920,203 Hepatitis C infections in Vietnam (including undiagnosed) [5].

Supporting Table S7: The projected mean breakpoint drug cost for 12-weeks' standard treatment resulting in the alternative strategy being cost-saving under the different cost scenarios

		Mean cost breakpoint (standard deviation)		
**Cost scenario**	**Value**	**Response-guided**	**Induction-maintenance**	**Peg-IFN+DAA**
Default assumptions	-	318 (48)	Cost saving[a]	703 (36)
The relative DAA drug cost of retreatment	Upper value	685 (86)	Cost saving[a]	834 (135)
Cost of the HCV-RNA viral load test	Lower value	188 (33)	Cost saving[a]	699 (34)
	Upper value	541 (75)	Cost saving[a]	713 (41)
Cost of Peg-IFN	Lower value	317 (49)	Cost saving[a]	499 (29)
	Upper value	316 (48)	Cost saving[a]	1271 (57)
The costs for patients accessing the treatment	Lower value	317 (49)	Cost saving[a]	499 (29)
	Upper value	350 (53)	Cost saving[a]	761 (39)
Number of treatment visits	Upper value	399 (54)	93 (1544)	745 (38)
Additional lost productivity due to adverse events for the Peg-IFN+DAA strategy	Upper value	317 (48)	Cost saving[a]	736 (37)
Non-drug costs scaling factor	Lower value	165 (28)	7 (1701)	625 (29)
	Upper value	620 (90)	Cost saving[a]	862 (50)

[a] *As the strategy is on average generating cost savings it does not have a corresponding cost breakpoint. The cost scenarios are described in Table 1. Costs are in 2023 US$ costs.*

Supporting Table S8: The projected strategy with the lowest cost per cure for different DAA drug costs under the cost scenarios

Cost scenario		Base-case DAA cost (US$963)	50	100	250	500	750	1000	1500
		DAA drug cost for a 12-week standard course							
Default assumptions	-	Induction-Maintenance	Induction-Maintenance	Induction-Maintenance	Induction-Maintenance	Induction-Maintenance	Induction-Maintenance	Induction-Maintenance	Peg-IFN+DAA
The relative DAA drug cost of the regimen used during a retreatment	Upper value	Induction-Maintenance	Induction-Maintenance	Induction-Maintenance	Induction-Maintenance	Induction-Maintenance	Induction-Maintenance	Induction-Maintenance	Peg-IFN+DAA
Cost of the HCV-RNA viral load test	Lower value	Response guided	Induction-Maintenance	Induction-Maintenance	Induction-Maintenance	Induction-Maintenance	Induction-Maintenance	Response guided	Peg-IFN+DAA
	Upper value	Induction-Maintenance	Induction-Maintenance	Induction-Maintenance	Induction-Maintenance	Induction-Maintenance	Induction-Maintenance	Induction-Maintenance	Peg-IFN+DAA
Cost of Peg-IFN	Lower value	Peg-IFN+DAA	Induction-Maintenance	Induction-Maintenance	Induction-Maintenance	Induction-Maintenance	Peg-IFN+DAA	Peg-IFN+DAA	Peg-IFN+DAA
	Upper value	Induction-Maintenance	Induction-Maintenance	Induction-Maintenance	Induction-Maintenance	Induction-Maintenance	Induction-Maintenance	Induction-Maintenance	Peg-IFN+DAA
The costs for patients accessing the treatment	Lower Value	Induction-Maintenance	Induction-Maintenance	Induction-Maintenance	Induction-Maintenance	Induction-Maintenance	Induction-Maintenance	Induction-Maintenance	Peg-IFN+DAA
	Upper value	Induction-Maintenance	Induction-Maintenance	Induction-Maintenance	Induction-Maintenance	Induction-Maintenance	Induction-Maintenance	Induction-Maintenance	Peg-IFN+DAA
Number of treatment visits	Upper value	Induction-Maintenance	Standard treatment	Induction-Maintenance	Induction-Maintenance	Induction-Maintenance	Induction-Maintenance	Induction-Maintenance	Peg-IFN+DAA
Additional lost productivity due to adverse events for the Peg-IFN+DAA strategy	Upper value	Induction Maintenance	Induction-Maintenance	Induction-Maintenance	Induction-Maintenance	Induction-Maintenance	Induction-Maintenance	Induction-Maintenance	Peg-IFN+DAA
Non-drug costs scaling factor	Lower Value	Response guided	Induction-Maintenance	Induction-Maintenance	Induction-Maintenance	Induction-Maintenance	Response guided	Response guided	Peg-IFN+DAA
	Upper value	Induction-Maintenance	Induction-Maintenance	Induction-Maintenance	Induction-Maintenance	Induction-Maintenance	Induction-Maintenance	Induction-Maintenance	Peg-IFN+DAA

The cost scenarios are described in Table 1.
The different strategies are highlighted in different colours.

Supporting Table S9: Mean cost per cure across the different cost scenario analyses

Cost scenarios	Mean cost per cure when using the lower value from the scenario analysis (standard deviation)				Mean cost per cure when using the upper value from the scenario analysis (standard deviation)			
	Standard	Response-guided	Induction maintenance	Peg-IFN+DAA	Standard	Response-guided	Induction maintenance	Peg-IFN+DAA
**The relative DAA drug cost of the retreatment regimen (upper range: 200% increase in the DAA drug cost for the retreatment regimen)**								
Drug cost for a 12-week standard course								
Base-case DAA cost (US$963)	NA	NA	NA	NA	1308	1105 (29)	1076 (81)	1145 (27)
50	NA	NA	NA	NA	383	465 (8)	370 (23)	790 (8)
100	NA	NA	NA	NA	434	500 (9)	409 (26)	809 (9)
250	NA	NA	NA	NA	586	605 (13)	525 (36)	867 (12)
500	NA	NA	NA	NA	839	781 (18)	718 (52)	965 (17)
750	NA	NA	NA	NA	1092	956 (24)	911 (68)	1062 (23)
1000	NA	NA	NA	NA	1345	1131 (30)	1104 (83)	1160 (28)
1500	NA	NA	NA	NA	1852	1482 (41)	1491 (115)	1355 (39)
**RNA viral load test cost (lower range: US$23.82, upper range: US$121.78)**								
Drug cost for a 12-week standard course								
Base-case DAA cost (US$963)	1236	994 (27)	1003 (73)	1070 (26)	1434	1301 (31)	1201 (85)	1277 (30)
50	311	353 (7)	298 (18)	714 (6)	509	661 (11)	496 (30)	921 (11)
100	362	388 (8)	337 (21)	734 (7)	560	696 (12)	535 (33)	941 (12)
250	514	494 (11)	453 (30)	792 (11)	712	801 (15)	650 (42)	999 (15)
500	767	669 (17)	646 (45)	890 (16)	965	976 (21)	843 (57)	1097 (20)
750	1020	844 (22)	838 (60)	987 (21)	1219	1152 (27)	1036 (72)	1194 (26)
1000	1273	1020 (28)	1031 (75)	1085 (27)	1472	1327 (32)	1229 (87)	1292 (31)
1500	1780	1371 (39)	1417 (105)	1280 (38)	1978	1678 (43)	1615 (117)	1487 (42)
**Weekly cost of Peg-IFN (lower range: US$52.34, upper range: US$172.37)**								
Drug cost for a 12-week standard course								
Base-case DAA cost (US$963)	1308	1106 (29)	1073 (73)	1018 (28)	1308	1106 (29)	1073 (67)	1499 (28)
50	383	465 (8)	370 (21)	662 (8)	383	465 (8)	369 (19)	1142 (8)
100	434	500 (9)	408 (24)	682 (9)	434	500 (9)	408 (22)	1162 (9)
250	586	605 (13)	524 (32)	740 (12)	586	605 (13)	524 (29)	1220 (12)
500	839	781 (18)	717 (46)	838 (18)	839	781 (18)	716 (42)	1318 (18)

750	1092	957 (24)	909 (61)	935 (23)	1092	956 (24)	909 (55)	1415 (23)
1000	1345	1132 (29)	1102 (75)	1033 (29)	1345	1132 (29)	1101 (68)	1513 (28)
1500	1852	1483 (41)	1487 (103)	1228 (40)	1852	1482 (41)	1487 (94)	1708 (39)
**The costs for patients accessing the treatment (lower range: US$11.59 per visit, upper range: US$37.04 per visit)**								
Drug cost for a 12-week standard course								
Base-case DAA cost (US$963)	1265	1051 (28)	1032 (69)	1059 (27)	1343	1151 (29)	1109 (78)	1216 (28)
50	341	410 (7)	327 (18)	703 (7)	418	510 (9)	405 (24)	860 (9)
100	391	445 (8)	366 (21)	723 (8)	469	545 (10)	443 (27)	880 (10)
250	543	550 (12)	482 (29)	781 (11)	620	651 (13)	559 (35)	938 (13)
500	796	726 (17)	675 (43)	879 (17)	874	826 (19)	752 (50)	1036 (18)
750	1050	901 (23)	867 (57)	976 (22)	1127	1001 (24)	945 (65)	1133 (24)
1000	1303	1077 (29)	1060 (71)	1074 (27)	1380	1177 (30)	1138 (80)	1231 (29)
1500	1809	1428 (40)	1446 (99)	1269 (38)	1887	1528 (41)	1523 (109)	1425 (40)
**Number of treatment visits (upper range: one additional visit for the three alternative strategies relative to standard treatment from what was assumed for the base-case)**								
Drug cost for a 12-week standard course								
Base-case DAA cost (US$963)	NA	NA	NA	NA	1308	1131 (28)	1074 (73)	1171 (28)
50	NA	NA	NA	NA	383	490 (8)	395 (21)	815 (8)
100	NA	NA	NA	NA	434	525 (9)	408 (24)	835 (9)
250	NA	NA	NA	NA	586	631 (13)	524 (32)	893 (12)
500	NA	NA	NA	NA	839	806 (18)	717 (46)	991 (18)
750	NA	NA	NA	NA	1092	982 (24)	909 (61)	1088 (23)
1000	NA	NA	NA	NA	1345	1157 (29)	1102 (75)	1185 (28)
1500	NA	NA	NA	NA	1852	1508 (40)	1488 (103)	1380 (39)
**Additional lost productivity due to adverse events for the Peg-IFN+DAA strategy (upper range: 39% of those taking Peg-IFN+DAA lose 2 days of work due to flu-like symptoms)**								
Drug cost for a 12-week standard course								
Base-case DAA cost (US$963)	NA	NA	NA	NA	1308	1106 (28)	1073 (68)	1166 (27)
50	NA	NA	NA	NA	383	465 (8)	370 (20)	810 (8)
100	NA	NA	NA	NA	434	500 (9)	408 (22)	829 (9)
250	NA	NA	NA	NA	586	605 (13)	524 (30)	888 (12)
500	NA	NA	NA	NA	839	781 (18)	716 (44)	985 (18)
750	NA	NA	NA	NA	1092	956 (24)	909 (57)	1083 (23)
1000	NA	NA	NA	NA	1345	1132 (29)	1102 (70)	1180 (28)
1500	NA	NA	NA	NA	1852	1483 (40)	1487 (97)	1375 (39)
**Non-drug cost (lower range: -50%, upper range: +100%)**								
Drug cost for a 12-week standard course								
Base-case DAA cost (US$963)	1142	894 (26)	909 (66)	931 (25)	1639	1531 (34)	1403 (79)	1575 (33)
50	217	253 (6)	205 (14)	575 (5)	714	890 (14)	700 (32)	1219 (13)
100	268	288 (7)	243 (17)	594 (6)	765	925 (15)	738 (34)	1238 (14)
250	420	393 (10)	359 (25)	653 (9)	917	1030 (18)	854 (42)	1297 (17)
500	673	569 (16)	552 (40)	750 (15)	1170	1206 (24)	1046 (55)	1394 (23)

750	926	744 (21)	744 (54)	848 (20)	1424	1381 (29)	1239 (68)	1492 (28)
1000	1180	920 (27)	937 (68)	945 (26)	1677	1556 (35)	1431 (81)	1589 (34)
1500	1686	1271 (38)	1323 (97)	1140 (36)	2183	1907 (46)	1816 (107)	1785 (44)

The cost scenarios are described in Table 1. Costs are in 2023 US$ costs.

Supporting Table S10: Difference in the mean cost per cure relative to the standard treatment stratified by different perspectives across the scenario analysis

**The relative DAA drug cost of retreatment regimen**	**Response guided**			**Induction maintenance**			**Peg-IFN+DAA**		
DAA drug cost for a 12-week standard course	Absolute overall difference - societal perspective (standard deviation)	Absolute difference - patient perspective (standard deviation)	Absolute difference - healthcare provider perspective (standard deviation)	Absolute overall difference - societal perspective (standard deviation)	Absolute difference - patient perspective (standard deviation)	Absolute difference - healthcare provider perspective (standard deviation)	Absolute overall difference - societal perspective (standard deviation)	Absolute difference - patient perspective (standard deviation)	Absolute difference - healthcare provider perspective (standard deviation)
50	87.41 (10.41)	33.24 (4.46)	54.18 (5.95)	-13.14 (27.99)	-6.48 (11.99)	-6.66 (16.00)	411.02 (10.12)	306.62 (4.34)	104.40 (5.79)
100	77.42 (13.76)	28.24 (6.13)	49.18 (7.63)	-25.44 (37.01)	-12.63 (16.49)	-12.81 (20.51)	384.26 (13.38)	293.24 (5.96)	91.02 (7.42)
250	47.44 (23.81)	13.25 (11.16)	34.19 (12.65)	-62.35 (64.04)	-31.09 (30.01)	-31.26 (34.03)	303.98 (23.16)	253.10 (10.85)	50.88 (12.31)
500	-2.52 (40.57)	-11.73 (19.54)	9.21 (21.03)	-123.86 (109.10)	-61.84 (52.54)	-62.02 (56.56)	170.18 (39.45)	186.20 (19.00)	-16.02 (20.45)
750	-52.48 (57.32)	-36.71 (27.91)	-15.77 (29.41)	-185.37 (154.16)	-92.60 (75.07)	-92.77 (79.09)	36.39 (55.75)	119.31 (27.15)	-82.92 (28.60)
1000	-102.44 (74.08)	-61.69 (36.29)	-40.75 (37.79)	-246.88 (199.22)	-123.35 (97.60)	-123.53 (101.62)	-97.41 (72.04)	52.41 (35.30)	-149.82 (36.75)
1500	-202.37 (107.59)	-111.66 (53.05)	-90.71 (54.54)	-369.91 (289.34)	-184.86 (142.66)	-185.04 (146.68)	-365.00 (104.64)	-81.39 (51.59)	-283.61 (53.04)
**Cost of the RNA viral load test - lower range**	**Response guided**			**Induction maintenance**			**Peg-IFN+DAA**		
DAA drug cost for a 12-week standard course	Absolute overall difference - societal perspective (standard deviation)	Absolute difference - patient perspective (standard deviation)	Absolute difference - healthcare provider perspective (standard deviation)	Absolute overall difference - societal perspective (standard deviation)	Absolute difference - patient perspective (standard deviation)	Absolute difference - healthcare provider perspective (standard deviation)	Absolute overall difference - societal perspective (standard deviation)	Absolute difference - patient perspective (standard deviation)	Absolute difference - healthcare provider perspective (standard deviation)
50	42.29 (6.59)	22.53 (3.02)	19.77 (3.56)	-12.81 (17.70)	-6.37 (8.12)	-6.44 (9.58)	403.46 (6.37)	303.78 (2.92)	99.68 (3.44)
100	26.73 (7.71)	14.75 (3.58)	11.98 (4.12)	-24.86 (20.70)	-12.39 (9.63)	-12.47 (11.08)	372.31 (7.45)	288.20 (3.46)	84.10 (3.98)
250	-19.96 (11.06)	-8.60 (5.26)	-11.36 (5.80)	-61.04 (29.71)	-30.48 (14.13)	-30.56 (15.58)	278.85 (10.69)	241.48 (5.08)	37.38 (5.60)
500	-97.78 (16.65)	-47.51 (8.05)	-50.27 (8.59)	-121.32 (44.72)	-60.63 (21.63)	-60.70 (23.08)	123.09 (16.08)	163.60 (7.78)	-40.50 (8.30)
750	-175.60 (22.23)	-86.42 (10.85)	-89.18 (11.39)	-181.61 (59.73)	-90.77 (29.14)	-90.84 (30.59)	-32.66 (21.48)	85.72 (10.48)	-118.38 (11.00)
1000	-253.41 (27.82)	-125.33 (13.64)	-128.09 (14.18)	-241.90 (74.74)	-120.91 (36.64)	-120.99 (38.09)	-188.42 (26.88)	7.84 (13.18)	-196.26 (13.70)
1500	-409.05 (38.99)	-203.14 (19.23)	-205.91 (19.77)	-362.48 (104.75)	-181.20 (51.65)	-181.28 (53.10)	-499.94 (37.68)	-147.92 (18.58)	-352.02 (19.10)
**Cost of the RNA viral load test – upper range**	**Response guided**			**Induction maintenance**			**Peg-IFN+DAA**		

DAA drug cost for a 12-week standard course	Absolute overall difference - societal perspective (standard deviation)	Absolute difference - patient perspective (standard deviation)	Absolute difference - healthcare provider perspective (standard deviation)	Absolute overall difference - societal perspective (standard deviation)	Absolute difference - patient perspective (standard deviation)	Absolute difference - healthcare provider perspective (standard deviation)	Absolute overall difference - societal perspective (standard deviation)	Absolute difference - patient perspective (standard deviation)	Absolute difference - healthcare provider perspective (standard deviation)
50	151.19 (10.96)	44.32 (3.90)	106.87 (7.07)	-13.41 (29.65)	-6.49 (10.54)	-6.92 (19.11)	412.00 (10.57)	305.49 (3.76)	106.51 (6.81)
100	135.63 (12.08)	36.54 (4.46)	99.09 (7.62)	-25.47 (32.67)	-12.52 (12.05)	-12.95 (20.62)	380.85 (11.65)	289.91 (4.30)	90.94 (7.35)
250	88.97 (15.43)	13.21 (6.13)	75.76 (9.30)	-61.64 (41.73)	-30.60 (16.59)	-31.04 (25.15)	287.39 (14.88)	243.18 (5.91)	44.21 (8.97)
500	11.19 (21.02)	-25.68 (8.93)	36.87 (12.09)	-121.93 (56.84)	-60.75 (24.14)	-61.18 (32.70)	131.63 (20.27)	165.30 (8.61)	-33.67 (11.66)
750	-66.58 (26.60)	-64.57 (11.72)	-2.02 (14.89)	-182.22 (71.94)	-90.89 (31.69)	-91.33 (40.25)	-24.13 (25.66)	87.42 (11.30)	-111.55 (14.35)
1000	-144.36 (32.19)	-103.45 (14.51)	-40.90 (17.68)	-242.51 (87.05)	-121.04 (39.24)	-121.47 (47.81)	-179.90 (31.04)	9.54 (13.99)	-189.43 (17.05)
1500	-299.91 (43.36)	-181.23 (20.10)	-118.68 (23.26)	-363.09 (117.26)	-181.33 (54.35)	-181.76 (62.91)	-491.42 (41.81)	-146.22 (19.38)	-345.20 (22.43)
**Cost of Peg-IFN – lower range**	**Response guided**			**Induction maintenance**			**Peg-IFN+DAA**		
DAA drug cost for a 12-week standard course	Absolute overall difference - societal perspective (standard deviation)	Absolute difference - patient perspective (standard deviation)	Absolute difference - healthcare provider perspective (standard deviation)	Absolute overall difference - societal perspective (standard deviation)	Absolute difference - patient perspective (standard deviation)	Absolute difference - healthcare provider perspective (standard deviation)	Absolute overall difference - societal perspective (standard deviation)	Absolute difference - patient perspective (standard deviation)	Absolute difference - healthcare provider perspective (standard deviation)
50	81.98 (8.20)	30.51 (3.35)	51.47 (4.85)	-13.38 (20.85)	-6.56 (8.52)	-6.83 (12.33)	279.42 (7.97)	215.42 (3.26)	64.00 (4.71)
100	66.44 (9.32)	22.74 (3.91)	43.70 (5.41)	-25.49 (23.70)	-12.61 (9.94)	-12.88 (13.75)	248.26 (9.06)	199.84 (3.80)	48.42 (5.26)
250	19.84 (12.68)	-0.56 (5.59)	20.40 (7.09)	-61.81 (32.24)	-30.77 (14.22)	-31.04 (18.03)	154.78 (12.33)	153.10 (5.44)	1.68 (6.89)
500	-57.84 (18.29)	-39.40 (8.39)	-18.44 (9.89)	-122.34 (46.49)	-61.04 (21.34)	-61.31 (25.15)	-1.02 (17.77)	75.20 (8.16)	-76.22 (9.61)
750	-135.52 (23.89)	-78.24 (11.20)	-57.28 (12.69)	-182.88 (60.73)	-91.30 (28.46)	-91.57 (32.27)	-156.82 (23.22)	-2.70 (10.88)	-154.12 (12.34)
1000	-213.20 (29.49)	-117.08 (14.00)	-96.12 (15.50)	-243.41 (74.98)	-121.57 (35.58)	-121.84 (39.39)	-312.62 (28.66)	-80.60 (13.60)	-232.02 (15.06)
1500	-368.56 (40.70)	-194.76 (19.60)	-173.80 (21.10)	-364.48 (103.46)	-182.10 (49.83)	-182.37 (53.64)	-624.23 (39.55)	-236.40 (19.05)	-387.82 (20.51)
**Cost of Peg-IFN – upper range**	**Response guided**			**Induction maintenance**			**Peg-IFN+DAA**		
DAA drug cost for a 12-week standard course	Absolute overall difference - societal perspective (standard deviation)	Absolute difference - patient perspective (standard deviation)	Absolute difference - healthcare provider perspective (standard deviation)	Absolute overall difference - societal perspective (standard deviation)	Absolute difference - patient perspective (standard deviation)	Absolute difference - healthcare provider perspective (standard deviation)	Absolute overall difference - societal perspective (standard deviation)	Absolute difference - patient perspective (standard deviation)	Absolute difference - healthcare provider perspective (standard deviation)
50	81.81 (8.19)	30.44 (3.35)	51.37 (4.84)	-13.52 (19.03)	-6.61 (7.78)	-6.91 (11.25)	759.31 (7.91)	551.30 (3.23)	208.00 (4.68)
100	66.25 (9.31)	22.66 (3.91)	43.59 (5.40)	-25.64 (21.63)	-12.67 (9.08)	-12.97 (12.55)	728.16 (8.99)	535.73 (3.77)	192.43 (5.22)
250	19.57 (12.67)	-0.68 (5.59)	20.25 (7.08)	-62.02 (29.43)	-30.86 (12.98)	-31.15 (16.46)	634.73 (12.24)	489.02 (5.40)	145.72 (6.84)
500	-58.22 (18.27)	-39.58 (8.39)	-18.65 (9.88)	-122.64 (42.44)	-61.17 (19.48)	-61.47 (22.96)	479.02 (17.64)	411.16 (8.10)	67.86 (9.54)
750	-136.02 (23.86)	-78.48 (11.18)	-57.54 (12.68)	-183.26 (55.44)	-91.49 (25.98)	-91.78 (29.46)	323.30 (23.05)	333.30 (10.80)	-10.00 (12.25)
1000	-213.82 (29.46)	-117.38 (13.98)	-96.44 (15.48)	-243.89 (68.44)	-121.80 (32.48)	-122.09 (35.96)	167.59 (28.46)	255.44 (13.51)	-87.85 (14.95)
1500	-369.41 (40.65)	-195.17 (19.58)	-174.24 (21.08)	-365.14 (94.45)	-182.42 (45.49)	-182.72 (48.96)	-143.84 (39.27)	99.73 (18.91)	-243.57 (20.36)
**The costs for patients accessing**	**Response guided**			**Induction maintenance**			**Peg-IFN+DAA**		

**the treatment – lower range**									
DAA drug cost for a 12-week standard course	Absolute overall difference - societal perspective (standard deviation)	Absolute difference - patient perspective (standard deviation)	Absolute difference - healthcare provider perspective (standard deviation)	Absolute overall difference - societal perspective (standard deviation)	Absolute difference - patient perspective (standard deviation)	Absolute difference - healthcare provider perspective (standard deviation)	Absolute overall difference - societal perspective (standard deviation)	Absolute difference - patient perspective (standard deviation)	Absolute difference - healthcare provider perspective (standard deviation)
50	69.07 (7.29)	17.65 (2.42)	51.42 (4.87)	-13.03 (18.13)	-6.33 (6.02)	-6.71 (12.11)	362.78 (6.95)	260.63 (2.31)	102.15 (4.64)
100	53.52 (8.41)	9.88 (2.98)	43.64 (5.43)	-25.11 (20.93)	-12.37 (7.42)	-12.75 (13.51)	331.62 (8.02)	245.05 (2.84)	86.57 (5.18)
250	6.88 (11.78)	-13.45 (4.67)	20.32 (7.12)	-61.35 (29.32)	-30.49 (11.62)	-30.86 (17.71)	238.16 (11.24)	198.32 (4.45)	39.83 (6.78)
500	-70.87 (17.41)	-52.32 (7.48)	-18.55 (9.93)	-121.75 (43.32)	-60.68 (18.61)	-61.06 (24.70)	82.38 (16.60)	120.43 (7.13)	-38.05 (9.47)
750	-148.61 (23.03)	-91.19 (10.29)	-57.42 (12.74)	-182.14 (57.31)	-90.88 (25.61)	-91.26 (31.70)	-73.40 (21.96)	42.54 (9.81)	-115.94 (12.15)
1000	-226.35 (28.65)	-130.06 (13.10)	-96.29 (15.55)	-242.54 (71.30)	-121.08 (32.61)	-121.46 (38.69)	-229.17 (27.32)	-35.34 (12.49)	-193.83 (14.83)
1500	-381.83 (39.90)	-207.80 (18.73)	-174.03 (21.17)	-363.33 (99.28)	-181.47 (46.60)	-181.85 (52.68)	-540.73 (38.04)	-191.12 (17.86)	-349.61 (20.19)
**The costs for patients accessing the treatment – upper range**	**Response guided**			**Induction maintenance**			**Peg-IFN+DAA**		
**DAA drug cost for a 12-week standard course**	Absolute overall difference - societal perspective (standard deviation)	Absolute difference - patient perspective (standard deviation)	Absolute difference - healthcare provider perspective (standard deviation)	Absolute overall difference - societal perspective (standard deviation)	Absolute difference - patient perspective (standard deviation)	Absolute difference - healthcare provider perspective (standard deviation)	Absolute overall difference - societal perspective (standard deviation)	Absolute difference - patient perspective (standard deviation)	Absolute difference - healthcare provider perspective (standard deviation)
**50**	92.30 (8.89)	40.94 (4.09)	51.36 (4.81)	-13.22 (23.65)	-6.56 (10.87)	-6.66 (12.78)	442.40 (8.57)	340.29 (3.94)	102.12 (4.63)
**100**	76.74 (10.01)	33.16 (4.64)	43.58 (5.36)	-25.29 (26.60)	-12.59 (12.34)	-12.70 (14.26)	411.24 (9.65)	324.71 (4.48)	86.53 (5.17)
**250**	30.06 (13.34)	9.82 (6.31)	20.24 (7.03)	-61.50 (35.46)	-30.69 (16.77)	-30.80 (18.69)	317.75 (12.86)	277.96 (6.08)	39.79 (6.78)
**500**	-47.75 (18.89)	-29.08 (9.09)	-18.67 (9.81)	-121.84 (50.23)	-60.87 (24.16)	-60.97 (26.07)	161.94 (18.21)	200.06 (8.76)	-38.11 (9.45)
**750**	-125.56 (24.45)	-67.99 (11.86)	-57.57 (12.58)	-182.19 (64.99)	-91.04 (31.54)	-91.15 (33.45)	6.13 (23.57)	122.15 (11.44)	-116.02 (12.13)
**1000**	-203.37 (30.00)	-106.89 (14.64)	-96.48 (15.36)	-242.53 (79.76)	-121.21 (38.92)	-121.32 (40.84)	-149.68 (28.92)	44.25 (14.11)	-193.92 (14.81)
**1500**	-358.99 (41.11)	-184.70 (20.19)	-174.29 (20.91)	-363.22 (109.29)	-181.56 (53.69)	-181.66 (55.60)	-461.30 (39.63)	-111.57 (19.47)	-349.74 (20.16)
**Number of treatment visits – upper range**	**Response guided**			**Induction maintenance**			**Peg-IFN+DAA**		
**DAA drug cost for a 12-week standard course**	Absolute overall difference - societal perspective (standard deviation)	Absolute difference - patient perspective (standard deviation)	Absolute difference - healthcare provider perspective (standard deviation)	Absolute overall difference - societal perspective (standard deviation)	Absolute difference - patient perspective (standard deviation)	Absolute difference - healthcare provider perspective (standard deviation)	Absolute overall difference - societal perspective (standard deviation)	Absolute difference - patient perspective (standard deviation)	Absolute difference - healthcare provider perspective (standard deviation)
50	107.37 (8.13)	56.00 (3.32)	51.37 (4.81)	12.27 (20.82)	19.04 (8.51)	-6.78 (12.31)	432.10 (7.92)	329.95 (3.24)	102.14 (4.69)
100	91.82 (9.24)	48.22 (3.88)	43.59 (5.36)	-25.39 (23.67)	-12.57 (9.93)	-12.82 (13.74)	400.94 (9.01)	314.38 (3.78)	86.57 (5.23)
250	45.14 (12.57)	24.88 (5.54)	20.25 (7.03)	-61.68 (32.21)	-30.71 (14.20)	-30.97 (18.01)	307.47 (12.26)	267.64 (5.40)	39.83 (6.85)
500	-32.66 (18.12)	-14.01 (8.32)	-18.64 (9.80)	-122.16 (46.43)	-60.95 (21.31)	-61.21 (25.12)	151.70 (17.67)	189.75 (8.11)	-38.06 (9.56)

750	-110.45 (23.68)	-52.91 (11.10)	-57.54 (12.58)	-182.63 (60.66)	-91.19 (28.43)	-91.44 (32.23)	-4.08 (23.08)	111.86 (10.82)	-115.95 (12.27)
1000	-188.25 (29.23)	-91.81 (13.87)	-96.44 (15.36)	-243.11 (74.89)	-121.43 (35.54)	-121.68 (39.35)	-159.86 (28.50)	33.97 (13.52)	-193.84 (14.97)
1500	-343.84 (40.33)	-169.60 (19.42)	-174.23 (20.91)	-364.06 (103.34)	-181.90 (49.77)	-182.16 (53.57)	-471.42 (39.32)	-121.81 (18.94)	-349.61 (20.39)
**Health insurance program contribution – upper range**	**Response guided**			**Induction maintenance**			**Peg-IFN+DAA**		
**DAA drug cost for a 12-week standard course**	Absolute overall difference - societal perspective (standard deviation)	Absolute difference - patient perspective (standard deviation)	Absolute difference - healthcare provider perspective (standard deviation)	Absolute overall difference - societal perspective (standard deviation)	Absolute difference - patient perspective (standard deviation)	Absolute difference - healthcare provider perspective (standard deviation)	Absolute overall difference - societal perspective (standard deviation)	Absolute difference - patient perspective (standard deviation)	Absolute difference - healthcare provider perspective (standard deviation)
50	81.53 (9.24)	22.60 (1.66)	58.94 (7.58)	-13.40 (22.69)	-0.27 (4.08)	-13.13 (18.61)	400.28 (8.90)	77.08 (1.60)	323.19 (7.30)
100	66.00 (10.36)	22.60 (1.66)	43.40 (8.70)	-25.49 (25.45)	-0.27 (4.08)	-25.22 (21.37)	369.13 (9.98)	77.08 (1.60)	292.04 (8.38)
250	19.39 (13.74)	22.60 (1.66)	-3.21 (12.08)	-61.74 (33.73)	-0.27 (4.08)	-61.48 (29.65)	275.68 (13.23)	77.08 (1.60)	198.59 (11.63)
500	-58.29 (19.36)	22.60 (1.66)	-80.89 (17.70)	-122.18 (47.54)	-0.27 (4.08)	-121.91 (43.46)	119.92 (18.64)	77.08 (1.60)	42.84 (17.04)
750	-135.98 (24.98)	22.60 (1.66)	-158.57 (23.32)	-182.61 (61.35)	-0.27 (4.08)	-182.34 (57.27)	-35.83 (24.06)	77.08 (1.60)	-112.91 (22.46)
1000	-213.66 (30.61)	22.60 (1.66)	-236.25 (28.95)	-243.04 (75.15)	-0.27 (4.08)	-242.77 (71.07)	-191.58 (29.47)	77.08 (1.60)	-268.66 (27.87)
1500	-369.02 (41.85)	22.60 (1.66)	-391.62 (40.19)	-363.91 (102.77)	-0.27 (4.08)	-363.64 (98.69)	-503.08 (40.30)	77.08 (1.60)	-580.16 (38.70)
**Additional lost productivity due to adverse events for the Peg-IFN+DAA strategy – upper range**	**Response guided**			**Induction maintenance**			**Peg-IFN+DAA**		
**DAA drug cost for a 12-week standard course**	Absolute overall difference - societal perspective (standard deviation)	Absolute difference - patient perspective (standard deviation)	Absolute difference - healthcare provider perspective (standard deviation)	Absolute overall difference - societal perspective (standard deviation)	Absolute difference - patient perspective (standard deviation)	Absolute difference - healthcare provider perspective (standard deviation)	Absolute overall difference - societal perspective (standard deviation)	Absolute difference - patient perspective (standard deviation)	Absolute difference - healthcare provider perspective (standard deviation)
50	81.86 (8.12)	30.46 (3.32)	51.40 (4.80)	-13.48 (19.55)	-6.60 (7.99)	-6.89 (11.56)	426.96 (7.85)	324.82 (3.21)	102.14 (4.64)
100	66.31 (9.23)	22.68 (3.87)	43.62 (5.36)	-25.60 (22.23)	-12.66 (9.33)	-12.95 (12.90)	395.81 (8.93)	309.24 (3.75)	86.56 (5.18)
250	19.65 (12.56)	-0.65 (5.54)	20.30 (7.02)	-61.97 (30.24)	-30.84 (13.34)	-31.13 (16.91)	302.34 (12.15)	262.51 (5.36)	39.83 (6.79)
500	-58.11 (18.11)	-39.53 (8.31)	-18.58 (9.80)	-122.57 (43.60)	-61.14 (20.02)	-61.43 (23.59)	146.56 (17.51)	184.62 (8.04)	-38.06 (9.47)
750	-135.87 (23.66)	-78.41 (11.09)	-57.47 (12.57)	-183.17 (56.96)	-91.44 (26.70)	-91.73 (30.27)	-9.22 (22.88)	106.73 (10.72)	-115.95 (12.16)
1000	-213.64 (29.21)	-117.29 (13.86)	-96.35 (15.35)	-243.78 (70.32)	-121.74 (33.38)	-122.03 (36.95)	-165.00 (28.25)	28.84 (13.41)	-193.84 (14.84)
1500	-369.16 (40.31)	-195.05 (19.41)	-174.11 (20.90)	-364.98 (97.04)	-182.35 (46.74)	-182.64 (50.31)	-476.56 (38.98)	-126.94 (18.77)	-349.62 (20.21)
**Non-drug costs scaling factor – lower range**	**Response guided**			**Induction maintenance**			**Peg-IFN+DAA**		
**DAA drug cost for a 12-week standard course**	Absolute overall difference - societal	Absolute difference - patient	Absolute difference - healthcare	Absolute overall difference - societal	Absolute difference - patient perspective (standard deviation)	Absolute difference - healthcare	Absolute overall difference - societal	Absolute difference - patient perspective (standard deviation)	Absolute difference - healthcare provider

	perspective (standard deviation)	perspective (standard deviation)	provider perspective (standard deviation)	perspective (standard deviation)		provider perspective (standard deviation)	perspective (standard deviation)		perspective (standard deviation)
50	35.23 (5.52)	11.77 (2.14)	23.46 (3.38)	-12.79 (13.91)	-6.30 (5.38)	-6.50 (8.53)	357.68 (5.22)	262.60 (2.02)	95.07 (3.20)
100	19.69 (6.65)	4.00 (2.70)	15.69 (3.95)	-24.88 (16.76)	-12.34 (6.81)	-12.54 (9.95)	326.52 (6.29)	247.03 (2.56)	79.49 (3.73)
250	-26.93 (10.04)	-19.31 (4.40)	-7.62 (5.64)	-61.13 (25.30)	-30.47 (11.08)	-30.67 (14.22)	233.06 (9.50)	200.30 (4.16)	32.76 (5.34)
500	-104.62 (15.69)	-58.16 (7.22)	-46.46 (8.47)	-121.57 (39.54)	-60.68 (18.20)	-60.88 (21.34)	77.29 (14.84)	122.41 (6.83)	-45.12 (8.01)
750	-182.32 (21.34)	-97.01 (10.05)	-85.31 (11.30)	-182.00 (53.78)	-90.90 (25.32)	-91.10 (28.46)	-78.48 (20.19)	44.53 (9.50)	-123.01 (10.68)
1000	-260.02 (26.99)	-135.86 (12.87)	-124.16 (14.12)	-242.43 (68.03)	-121.11 (32.44)	-121.31 (35.58)	-234.25 (25.53)	-33.36 (12.18)	-200.89 (13.35)
1500	-415.42 (38.30)	-213.56 (18.52)	-201.86 (19.77)	-363.29 (96.51)	-181.54 (46.68)	-181.74 (49.82)	-545.79 (36.22)	-189.13 (17.52)	-356.66 (18.70)
**Non-drug costs scaling factor – upper range**	**Response guided**			**Induction maintenance**			**Peg-IFN+DAA**		
**DAA drug cost for a 12-week standard course**	Absolute overall difference - societal perspective (standard deviation)	Absolute difference - patient perspective (standard deviation)	Absolute difference - healthcare provider perspective (standard deviation)	Absolute overall difference - societal perspective (standard deviation)	Absolute difference - patient perspective (standard deviation)	Absolute difference - healthcare provider perspective (standard deviation)	Absolute overall difference - societal perspective (standard deviation)	Absolute difference - patient perspective (standard deviation)	Absolute difference - healthcare provider perspective (standard deviation)
50	175.24 (13.60)	67.89 (5.79)	107.35 (7.81)	-14.81 (31.53)	-7.19 (13.43)	-7.62 (18.10)	504.43 (13.12)	388.03 (5.59)	116.40 (7.53)
100	159.68 (14.72)	60.11 (6.35)	99.58 (8.36)	-26.95 (34.12)	-13.26 (14.73)	-13.69 (19.39)	473.29 (14.19)	372.46 (6.13)	100.82 (8.07)
250	113.03 (18.06)	36.78 (8.03)	76.25 (10.04)	-63.38 (41.88)	-31.48 (18.61)	-31.91 (23.27)	379.86 (17.42)	325.75 (7.74)	54.11 (9.68)
500	35.27 (23.64)	-2.10 (10.82)	37.37 (12.83)	-124.10 (54.82)	-61.84 (25.08)	-62.27 (29.74)	224.15 (22.80)	247.90 (10.43)	-23.74 (12.37)
750	-42.49 (29.22)	-40.98 (13.61)	-1.51 (15.62)	-184.82 (67.75)	-92.19 (31.54)	-92.62 (36.21)	68.44 (28.19)	170.04 (13.12)	-101.60 (15.06)
1000	-120.25 (34.80)	-79.86 (16.40)	-40.39 (18.41)	-245.53 (80.69)	-122.55 (38.01)	-122.98 (42.68)	-87.26 (33.57)	92.19 (15.81)	-179.45 (17.75)
1500	-275.78 (45.96)	-157.62 (21.98)	-118.16 (23.99)	-366.97 (106.56)	-183.27 (50.95)	-183.70 (55.61)	-398.68 (44.33)	-63.52 (21.20)	-335.16 (23.14)

The cost scenarios are described in Table 1. Costs are in 2023 US$ costs.

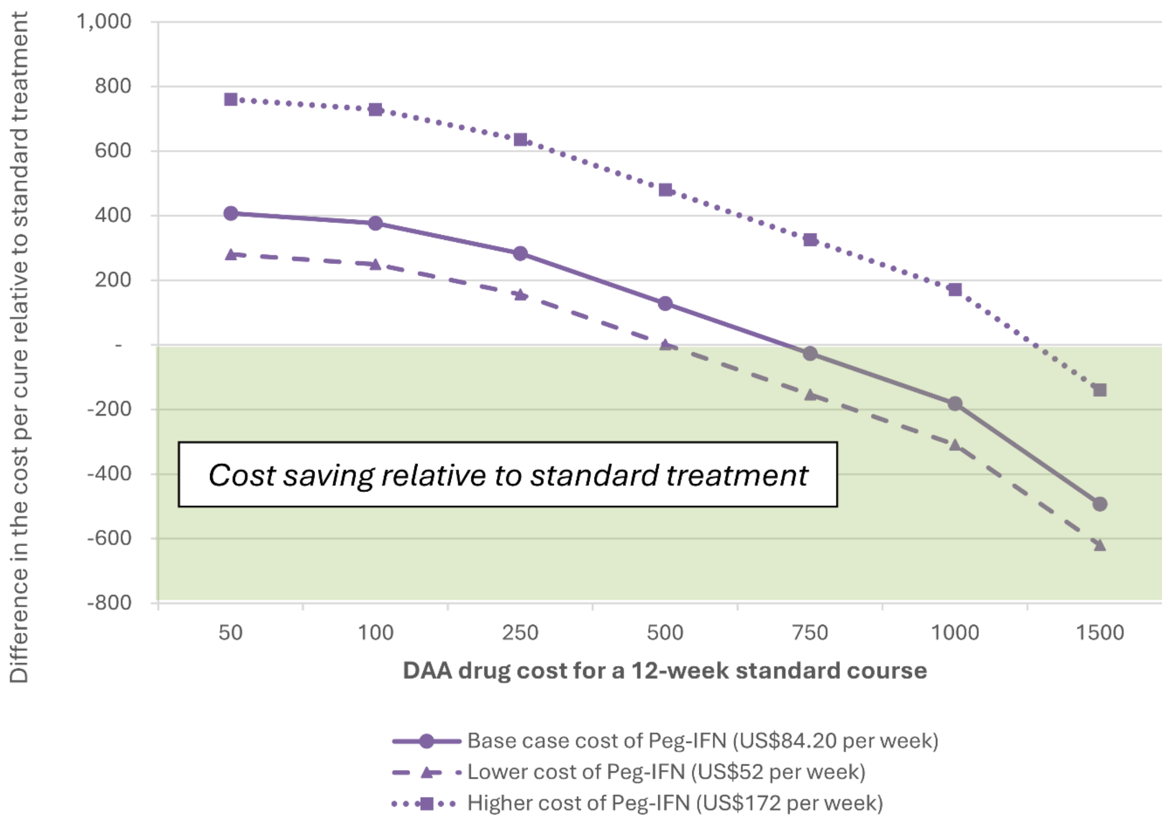

Supporting Figure S3: Scenario analysis regarding the weekly cost of Peg-IFN. *The green area highlights where the difference in the cost per cure relative to standard treatment is negative i.e. the strategy generates cost savings. The scenarios are described in Table 1. Costs are in 2023 US$ costs.*

References

1. General Statistics Office of Vietnam. Available from: https://www.gso.gov.vn/en/homepage/.
2. Nguyen HA. Health Economics Evaluation for Hepatitis C in Vietnam 2023.
3. Clinton Health Access Initiative. HCV Market Intelligence Report: Issue 3. 2023.
4. Cooke GS, Hung LM, Flower B, McCabe L, Hang VTK, Thu VT, et al. Treatment options to support the elimination of hepatitis C: an open-label, factorial, randomised controlled non-inferiority trial. The Lancet. 2025. doi: https://doi.org/10.1016/S0140-6736(25)00097-2.
5. Polaris Dashboard (Vietnam). Available from: https://cdafound.org/polaris/dashboard/.
