# Supplementary material for: Economic evaluation of alternative hepatitis C treatment options: a post hoc analysis of the VIETNARMS trial
Source: eClinicalMedicine. 2026 May 7;95:103969. doi: 10.1016/j.eclinm.2026.103969 (PMC13156567; doi:10.1016/j.eclinm.2026.103969)
Supplement: SEARCH investigators [file mmc3.docx]

**List of SEARCH investigators (Alphabetical order) that are not covered in the named co-author list:**

| **First Name** | **Surnames** |
| --- | --- |
| Azim | Ansari |
| Eleanor | Barnes |
| Dang Thi | Bich |
| Dao Bach | Khoa |
| Jeremy N. | Day |
| Barnaby | Flower |
| Leanne | McCabe |
| Richard M. | Hoglund |
| Evelyne | Kestelyn |
| Cherry | Kingsley |
| Chau | Le Ngoc |
| Le Thanh | Phuong |
| Le Thi | Thao |
| Nguyen Bao | Tran |
| An | Nguyen Chau |
| Nguyen Kim | Tuyen |
| Nguyen Ngoc | Phuong |
| Sarah | Pett |
| Pham Ngoc | Thach |
| Motiur | Rahman |
| David | Smith |
| Joel | Tarning |
| Rogier | Van Doorn |
| Vo Minh | Quang |
| Vo Thi | Thu |
| Vu Kim | Hang |
| Vu Thu | Huong |
