# Supplementary material for: Economic evaluation of alternative hepatitis C treatment options: a post hoc analysis of the VIETNARMS trial
Source: eClinicalMedicine. 2026 May 7;95:103969. doi: 10.1016/j.eclinm.2026.103969 (PMC13156567; doi:10.1016/j.eclinm.2026.103969)
Supplement: Supplementary file captions [file mmc4.docx]

**Supplementary file captions**

Supporting File 1. Supplementary Tables and Figures and further methodological information

Supporting File 2: The Excel-based model used for the calculations

Supporting File 3: CHEERS checklist

Supporting File 4: List of SEARCH investigators
